# Supplementary material for: Study protocol for an international, multicentre stepped-wedge cluster randomised trial to evaluate the impact of a digital antimicrobial stewardship smartphone application
Source: BMJ Open. 2020 Jun 4;10(6):e033640. doi: 10.1136/bmjopen-2019-033640 (PMC7279644; doi:10.1136/bmjopen-2019-033640)
Supplement: Supplementary data [file bmjopen-2019-033640supp001.pdf]

Appendix 1

CRF for appropriate antimicrobial drug use

1. Age of patient on date on study inclusion?
2. Gender of patient?
3. Allergy listed / specified?
    a. Listed & specified
    b. Listed without specification
    c. Not listed
    d. Not assessable
4. Date of hospital admission?
5. To which ward is patient admitted?
6. Date of admission to this ward unit?
7. Date of discharge from this ward/unit?
8. Indication listed or documented in admission note within 24 hours after starting antimicrobial drugs?

9.1.1 On which ward was the first antimicrobial drug prescribed?
9.1.2 What antimicrobial drug was prescribed?
9.1.3 On what date was the antimicrobial drug prescribed?
9.1.4 At what time was the antimicrobial drug prescribed?
9.1.5 Initial route of first antimicrobial drug prescribed?
9.1.6 Were there more than one therapeutic antimicrobial drug prescribed?

10. Correct choice of antimicrobial drugs according to the guidelines at time of prescribing according to indication?
    a. Fully concordant
    b. Partially concordant
    c. Not concordant
    d. No guideline available for specific indication
    e. Not assessable (no indication written)
11. Dosing regimen correct for all of the given antimicrobial drugs (adjusted for renal function too)?
12. Initial route of administration correct according to local guideline of all antimicrobial drugs?
13. Blood cultures performed?
14. Cultures or relevant microbial testing from suspected sites of infection performed according to local guideline (excluding blood cultures)?

15. PCR/Culture taken during this admission positive for C. difficile or MDRO (MRSA, ESBL-E, CPE, VRE, multidrug resistant P. aeruginosa)?
16. De-escalation from empirical to pathogen-directed therapy within 5 days after starting treatment for all antimicrobial drugs?
17. Switch IV to PO after 48-96h if clinically appropriate according to local guidelines?
18. Duration of antibiotic plan documented in medical chart? (between admission to this ward and day 5)
19. Duration of actual given antimicrobial drugs in accordance to local guidelines?
20. Therapeutic drug monitoring (TDM) according to local guidelines (aminoglycosides, glycopeptides, azoles) for all antimicrobial drugs?
21. Did the patient receive antimicrobial drugs <30 days before start of (empirical/targeted) treatment?
22. Have any of the following complications occurred?
    a. In-hospital mortality within 30 days after hospital admission (all cause)
    b. Transfer to intermediate care
    c. Transfer to ICU
    d. Unplanned readmission within 30 days after this admission
    e. Combination of answers above
    f. No
23. Is there an ID specialist / clinical microbiologist consulted for this treatment episode?
    a. Yes, in the first 5 days after start of antimicrobial treatment
    b. Yes, after 5 days of start antimicrobial treatment
    c. No
    d. Not assessable
