# Supplementary material for: Study protocol for an international, multicentre stepped-wedge cluster randomised trial to evaluate the impact of a digital antimicrobial stewardship smartphone application
Source: BMJ Open. 2020 Jun 4;10(6):e033640. doi: 10.1136/bmjopen-2019-033640 (PMC7279644; doi:10.1136/bmjopen-2019-033640)
Supplement: Supplementary data [file bmjopen-2019-033640supp002.pdf]

Appendix 2: Post-intervention questionnaire

For each of the questions below, circle the response that characterizes best how you feel about each statement,

where: 1 = Strongly disagree, 2 = Disagree, 3 = Neither disagree nor agree, 4 = Agree, 5 = Strongly agree

	Strongly disagree	Disagree	Neither agree nor disagree	Agree	Strongly agree
1. This app explains clearly what I have to do and in which order	1	2	3	4	5
2. This app includes every step of appropriate antibiotic use in the hospital	1	2	3	4	5
3. This app is too complex for use in daily practice	1	2	3	4	5
4. Guidelines in the app were accessible	1	2	3	4	5
5. Guidelines in the app were consistent with other guidelines	1	2	3	4	5
6. This app fits in current practices	1	2	3	4	5
7. The benefits of using the app are clear	1	2	3	4	5
8. This app is feasible for all my patients who receive IV antibiotics	1	2	3	4	5
9. This app is a threat to my professional autonomy	1	2	3	4	5

10. I already knew the guidelines in the app	1	2	3	4	5
11. I trust the guidelines in the app	1	2	3	4	5

	Strongly disagree	Disagree	Neither agree nor disagree	Agree	Strongly agree
12. I agree with the guidelines in the app	1	2	3	4	5
13. I have a positive attitude towards guidelines in general	1	2	3	4	5
14. I expect that this app will improve the quality of my antibiotic prescriptions	1	2	3	4	5
15. I could not observe benefits of using the app	1	2	3	4	5
16. It is part of my job to use this app	1	2	3	4	5
17. I intend to use the app to assess the guidelines	1	2	3	4	5
18. I am capable of using this app	1	2	3	4	5
19. It does not take a lot of effort to use this app	1	2	3	4	5
20. I was able to try the app before using it for actual prescriptions	1	2	3	4	5
21. I have enough knowledge and expertise to use the app adequately	1	2	3	4	5

22. I have good previous experiences with working with an app	1	2	3	4	5
23. Colleagues will support me to use this	1	2	3	4	5
24. Supervisors will support me to use this app	1	2	3	4	5

	Strongly disagree	Disagree	Neither agree nor disagree	Agree	Strongly agree
25. Nurses will support me to use this app	1	2	3	4	5
26. Colleagues will use this app	1	2	3	4	5
27. Colleagues will expect me to use the app	1	2	3	4	5
28. Supervisors will expect me to use the app	1	2	3	4	5
29. Nurses will expect me to use the app	1	2	3	4	5
30. Patient will be satisfied that this app is being used	1	2	3	4	5
31. I expect that this app will improve the patient's antibiotic treatment	1	2	3	4	5
32. There are enough financial resources to use the app as it is meant to be used	1	2	3	4	5
33. I can use the app while working with the electronic health records	1	2	3	4	5

system					
34. External support is needed to implement the app on organizational level	1	2	3	4	5
