# Supplementary material for: Study protocol for an international, multicentre stepped-wedge cluster randomised trial to evaluate the impact of a digital antimicrobial stewardship smartphone application
Source: BMJ Open. 2020 Jun 4;10(6):e033640. doi: 10.1136/bmjopen-2019-033640 (PMC7279644; doi:10.1136/bmjopen-2019-033640)
Supplement: Author's manuscript [file bmjopen-2019-033640.draft_revisions.pdf]

BMJ Open

BMJ Open is committed to open peer review. As part of this commitment we make the peer review history of every article we publish publicly available.

When an article is published we post the peer reviewers' comments and the authors' responses online. We also post the versions of the paper that were used during peer review. These are the versions that the peer review comments apply to.

The versions of the paper that follow are the versions that were submitted during the peer review process. They are not the versions of record or the final published versions. They should not be cited or distributed as the published version of this manuscript.

BMJ Open is an open access journal and the full, final, typeset and author-corrected version of record of the manuscript is available on our site with no access controls, subscription charges or pay-per-view fees (http://bmjopen.bmj.com).

If you have any questions on BMJ Open's open peer review process please email info.bmjopen@bmj.com

BMJ Open

Study protocol for an international, multicenter stepped-wedge cluster randomized trial to evaluate the impact of a digital antimicrobial stewardship smartphone application

Journal:	*BMJ Open*
Manuscript ID	bmjopen-2019-033640
Article Type:	Protocol
Date Submitted by the Author:	14-Aug-2019

Complete List of Authors:	Helou, R.I.; Erasmus Medical Center, Department of Medical Microbiology and Infectious Diseases<br>Catho, Gaud ; Hopitaux Universitaires de Geneve, Department of Infectious Diseases<br>Peyravi Latif, Annabel; Uppsala University Hospital, Department of Infectious Diseases<br>Mouton, Johan; Erasmus Medical Center, Department of Medical Microbiology and Infectious Diseases<br>Hulscher, M; Radboud University Medical Center, Scientific Center for Quality of Healthcare (IQ healthcare), Radboud Institute for Health Sciences<br>Teerenstra, Steven; Radboud University Medical Center, Department for Health Evidence, Radboud Institute for Health Sciences<br>Conly, John; University of Calgary and Alberta Health Services, Department of Medicine<br>Huttner, Benedikt D; Hopitaux Universitaires de Geneve, Department of Infectious Diseases<br>Tängdén, Thomas; Uppsala University Hospital, Department of Infectious Diseases<br>Verbon, Annelies ; Erasmus Medical Center, Department of Medical Microbiology and Infectious Diseases

Keywords:	INFECTIOUS DISEASES, MICROBIOLOGY, Protocols & guidelines < HEALTH SERVICES ADMINISTRATION & MANAGEMENT, Information technology < BIOTECHNOLOGY & BIOINFORMATICS, THERAPEUTICS

SCHOLARONE™
Manuscripts

I, the Submitting Author has the right to grant and does grant on behalf of all authors of the Work (as defined in the below author licence), an exclusive licence and/or a non-exclusive licence for contributions from authors who are: i) UK Crown employees; ii) where BMJ has agreed a CC-BY licence shall apply, and/or iii) in accordance with the terms applicable for US Federal Government officers or employees acting as part of their official duties; on a worldwide, perpetual, irrevocable, royalty-free basis to BMJ Publishing Group Ltd ("BMJ") its licensees and where the relevant Journal is co-owned by BMJ to the co-owners of the Journal, to publish the Work in this journal and any other BMJ products and to exploit all rights, as set out in our licence.

The Submitting Author accepts and understands that any supply made under these terms is made by BMJ to the Submitting Author unless you are acting as an employee on behalf of your employer or a postgraduate student of an affiliated institution which is paying any applicable article publishing charge ("APC") for Open Access articles. Where the Submitting Author wishes to make the Work available on an Open Access basis (and intends to pay the relevant APC), the terms of reuse of such Open Access shall be governed by a Creative Commons licence – details of these licences and which Creative Commons licence will apply to this Work are set out in our licence referred to above.

Other than as permitted in any relevant BMJ Author's Self Archiving Policies, I confirm this Work has not been accepted for publication elsewhere, is not being considered for publication elsewhere and does not duplicate material already published. I confirm all authors consent to publication of this Work and authorise the granting of this licence.

For peer review only

Study protocol for an international, multicenter stepped-wedge cluster randomized trial to evaluate the impact of a digital antimicrobial stewardship smartphone application

For peer review only

R.I. Helou[1], G. Catho[2], A. Peyravi Latif[3], J.W. Mouton[1], M.E.J.L. Hulscher[4], S. Teerenstra[5], J. Conly[6], B. D. Huttner[2], T. Tängdén[3], A. Verbon[1*]

1) Department of Medical Microbiology and Infectious Diseases, Erasmus Medical Center, Rotterdam, The Netherlands
2) Department of Infectious Diseases, Geneva University Hospitals and Faculty of Medicine, Geneva, Switzerland
3) Department of Infectious Diseases, Uppsala University Hospital, Uppsala, Sweden
4) Scientific Center for Quality of Healthcare (IQ healthcare), Radboud Institute for Health Sciences, Radboud University Medical Center
5) Department for Health Evidence, Radboud Institute for Health Sciences, Radboud University Medical Center
6) Department of Medicine, University of Calgary and Alberta Health Services, Calgary, Alberta, Canada

*) corresponding author

Abstract

Introduction With the widespread use of electronic health records (EHR) and handheld electronic devices in hospitals, informatics-based antimicrobial stewardship (AMS) interventions hold great promise as tools to promote appropriate antimicrobial drug prescribing. However, more research is needed to evaluate their optimal design and impact on quantity and quality of antimicrobial prescribing.

Methods and analysis Use of smartphone-based digital stewardship applications (app) with local guideline directed empirical antimicrobial use by physicians will be compared to antimicrobial prescription as per usual as primary outcome in three hospitals in the Netherlands, Sweden and Switzerland. Secondary outcomes will include antimicrobial use metrics, clinical and process outcomes. A multicenter stepped-wedge cluster randomized trial will randomize entities defined as wards or specialty regarding time of introduction of the intervention. By including 24 hospital entities with 7 measurement periods in which the primary outcome will be measured in 15 patients per cluster (ICC=0.1), we have a power of 81% to detect an improvement from 60% to 70% appropriate antimicrobial use. To account for drop-out or a lower inclusion rate, we will include 36 entities. At participating wards, patients of at least 18 years of age using antimicrobials will be included. After a baseline period of 2-week measurements, 6 periods of 4 weeks will follow in which the intervention is introduced in 6 wards (in 3 hospitals) until all 36 wards have implemented the intervention. Thereafter, we allow use of the app by everyone, and evaluate the sustainability of the app use 6 months later.

Ethics and dissemination This protocol will be initiated following approval by the institutional review board (IRB) of each participating center. Results will be disseminated via media, to healthcare professionals via professional training and meetings and to researchers via conferences and publications.

Registration details This study is registered on ClinicalTrials.gov under identifier NCT03793946.

For peer review only

Strengths and limitations of this study

- This study will be an international randomized, multi-center, clinical trial using a rigorous design and methodology to evaluate the impact of an AMS smartphone app for the hospital setting
- The app can easily be adapted to local guidelines which is a key feature for potential future use in other settings
- The primary outcome is appropriate empiric antimicrobial therapy. The study is conducted in three hospitals with relatively high rates of appropriate antibiotic use, which may theoretically lead to a higher risk of a negative trial
- In this cluster randomized trial, wards/specialties are the unit of randomization and there is a risk of contamination through physicians changing between wards or among specialties, although this risk is lower than for a single-center randomized trial. Restricted access to the software and tracking of app use will be applied to monitor possible contamination.

Introduction

Antimicrobial drugs are an indispensable part of modern medicine, which depends on effective drugs for prophylaxis and treatment. Yet, only around 40-70% of empiric antimicrobial regimens are considered appropriate with regard to their indication, the choice of agent, dosing or duration.(1-4) Inappropriate antimicrobial use enhances the risks of treatment failure and side effects in the individual patient, and accelerates selection and transmission of antimicrobial-resistant pathogens in health care settings and the community.(5) Many antimicrobial stewardship (AMS) interventions have been shown to increase appropriateness of antimicrobial drug prescribing.(6)

A software application (app), which is compatible with mobile electronic devices such as smartphones and tablets, and increases the availability of existing national and local antibiotic guidelines plus local antibiograms, may improve antimicrobial prescribing, reduce medication errors and ultimately improve patient outcome. Due to its highly customizable nature, a digital app has the potential to become an effective AMS tool in all countries.

Mobile-software apps seem particularly interesting for the purpose of AMS, since they do not require access to electronic health records or computerized physician order entry systems, that are costly, difficult to maintain and not easily customizable. Most healthcare workers own a smartphone and often use it to access information to assist in their treatment decisions.(7, 8)

For peer review only

The vast majority of available apps developed to support prescription of medicines are limited to knowledge content and do not provide decision support algorithms. Further, they have rarely been evaluated in clinical trials.(9) To date, most reported digital AMS interventions are single-center studies with limited internal validity due to methodological limitations and uncertain external validity.(4, 10, 11)

In the present study, we will evaluate the implementation and impact of a smartphone-based software application (the AB-Assistant, Spectrum, Calgary, Canada) adapted to local conditions in an international clinical trial using a stepped-wedge cluster randomized design and appropriateness of empiric antimicrobial prescribing as the primary outcome.

Methods and analysis

Setting

This study will be conducted in three academic, tertiary care hospitals in Europe: Erasmus University Medical Center, Rotterdam, the Netherlands; Uppsala University Hospital, Uppsala, Sweden; and Geneva University Hospitals, Geneva, Switzerland (Table 1).

Study design

We plan to conduct an international, multicenter, stepped wedge cluster randomized trial to assess whether making a smartphone application for AMS (Spectrum) can improve the appropriateness of antimicrobial prescribing in hospitalized patients. Table 2 illustrates the study question according to the PICOT framework.

In total, 36 medical and surgical entities (defined as wards (Sweden) or specialties (Switzerland, The Netherlands) will be included in the study using a stepwise design with 6 entities (2 entities per participating hospital) 4-weekly at 6 introduction moments (Figure 1). The primary outcome is appropriateness of empiric antimicrobial prescriptions assessed prior to implementation and up to 12 months after the intervention (after 6 months free use of the app).

The entities will be randomized as to the time period of making the app available (see intervention below). The order of implementation will be determined by a computer-generated list of random numbers programmed by a statistician otherwise not involved in the trial and AMS activities. Randomization will be stratified by type of specialty (medicine, surgery etc.). After a baseline period of 2-week measurements, 6 periods of 4 weeks will follow in which the

For peer review only

intervention is introduced in 6 entities (in 3 hospitals) until all entities have implemented the intervention. We plan to include 36 entities in total (see sample size calculation below) during the 6 introduction moments and at the end of the inclusion time we will allow the use of the app by everyone, also wards/specialties not included in the study. In this way, the trend in appropriate antimicrobial use can be monitored per ward/specialty and the decrease of antimicrobial use and appropriateness of empirical antimicrobial therapy can be followed during the stepwise implementation.(12) At 12 months (after 6 months free use of the app) we will have a 2-week measurement period to evaluate the sustainability of the intervention.

Justification of the stepped wedge cluster randomized study design

The stepped-wedge cluster randomized design was chosen after considering several advantages and disadvantages of randomization at the patient, physician, specialty and ward level. Patients are the "classic unit" of randomization and outcomes are often assessed at the patient level. However, there is a large risk of contamination of the intervention since one patient is often taken care of by many different physicians (see Figure 2). Furthermore, the study intervention targets physicians and not patients, therefore precluding randomization at the patient level as an option. Randomization at the physician-level is, however, equally problematic since prescribing decisions in hospitals are often made by teams of physicians, making it difficult to assign a decision to a specific individual physician and introducing the risk of contamination between physicians within a team. We therefore decided to use the wards/specialty as entity of randomization since it minimizes the risk of contamination and reflects "real-life" antimicrobial stewardship in hospitals where interventions are usually implemented at a ward/specialty level.

The stepped-wedge design, which involves the randomized and sequential rollout of the intervention to clusters over time, was chosen because it can model and adjust for underlying temporal trends and results in all clusters having access to the intervention at the end of the rollout period which seems desirable from an antimicrobial stewardship perspective.(13)

Study population

In our study, the intervention will be targeted at physicians that prescribe antimicrobials. To avoid possible contamination, the prescribers will be randomized at the ward/specialty level and not individually (see above). Process parameters will be determined in physicians who have been randomized to use the app per the process indicated above. Outcome parameters such as appropriate antimicrobial use will be determined at the patient level at participating wards

For peer review only

(control and intervention) who meet the following criteria: being a medical or surgical specialty patient at least 18 years of age for whom antimicrobial drugs are prescribed.

Intervention

The Spectrum application, available for iOS and Android and on the web, has been developed in Canada (www.spectrum.app) and is now being used in over 50 hospitals, mainly in Canada and the United States of America. The app has been developed with the help of human factor experts and integrates multiple resources into a unified user-friendly interface for use at point-of-care to improve antimicrobial prescribing (Figure 3).

For the purpose of this study the app and the content management system were extended to also support languages other than English (French, Dutch, Swedish). App content such as local guidelines, information regarding antimicrobial drugs, and micro-organisms of each hospital was entered by investigators of the Dutch, Swiss and Swedish hospitals (RH, GC, BH, APL). The entered content was checked again by other investigators before the start of the study.

The allocation of the timing to introduce the app will be concealed to the participating cluster up until 2 weeks before initiating use of the intervention. At the start of the intervention physicians on the intervention ward(s) will be informed about the function and content of the app. During the trial, only physicians on the intervention wards will be whitelisted to be able to use the app. Access to the app will be centrally withdrawn if a physician leaves the intervention ward (for example to work on a different (i.e. non-intervention) ward). The conventional route of guidelines (e.g. portable document formats (PDF), website or printed booklet) will still be available at all times. Physicians can freely choose between the app and the conventional route. Physicians on control wards will only be able to access the guidelines using the conventional route.

Patient and Public Involvement

No patients or public have been included in the study design.

Outcome parameters

Outcome parameters (Table 3) will be monitored using a digital CRF (Appendix 1) in the software application OpenClinica. (14)

Primary outcome parameter

Appropriateness of empiric antimicrobial therapy expressed as number and percentage of appropriate treatment episodes compared to all assessed treatment episodes.

For peer review only

Definitions for the primary outcome are:

- antimicrobial drugs assessed are ATC classes J01, J02, J03, J04, J05 (excluding anti-HIV drugs), P01, P02 and P03.
- treatment episodes are defined as the duration of treatment given for a specific infectious episode with gaps of treatment of no longer than one day.
- treatments are antimicrobials started during the measurement period at the participating entity. If a treatment was started within 24 hours before transfer in the emergency room or other ward and has not been changed during the first 24 hour stay at the ward, the treatment will be considered in accordance with the choice of the ward physician.
- treatments are in patients either newly admitted to the participating entities or already hospitalized during the pre-intervention period.
- appropriateness of empiric therapy includes choice, route and the dose of the antimicrobial drugs.
- appropriateness of therapy is in concordance with local treatment guidelines. If no guidelines are available, or if the treatment deviates from the guidelines or if treatment is guideline concordant but treatment should have deviated from the guideline (e.g. because of allergies, colonization with multidrug-resistant bacteria, other microbiological results, interactions, comorbidities or recent exposure to antimicrobials), cases will be discussed among 2 independent local infectious disease (ID) physicians. If they do not agree a third independent local ID physician will be consulted to adjudicate.
- for each patient only the first treatment episode during the measurement period will be evaluated. For patients treated for >1 infection (e.g. pneumonia and *Clostridium difficile* infection) appropriateness will be assessed for all infections combined (e.g. if treatment is appropriate for *C. difficile* infection but inappropriate for pneumonia the treatment would be categorized as inappropriate).

Secondary outcome parameters

- Total prescription of antimicrobial drugs on the cluster-level expressed as DDD and DOT denominated by patient-days and admission; subcategories by type of antimicrobial (antibiotic, antifungal, etc.), WHO AWaRE categories and restricted antimicrobial drugs (Table 3).

For peer review only

- Clinical secondary outcomes: length of hospital stay, in hospital mortality and readmissions within 30 days after discharge, incidence/prevalence of key AMR pathogens such as *C. difficile*.
- Process parameters of the intervention: penetration of the AB-assistant (total users and number of use of app).

Alongside the RCT, an uptake analysis will be performed to assess actual use of the app as well as an evaluation of use.

Uptake analysis

A number of parameters regarding use of the app will be collected and made available on a secure dashboard to authorized users at each site. These include number of monthly users and monthly sessions, number of times each antimicrobial, pathogen or clinical pathway screen is viewed, use by hospital, department, and medical specialism. These data will enable in-app behavior to be correlated with actual prescribing behavior on ward level and site-wide basis, thereby strengthening the association of the intervention to observed outcomes.

Evaluation of use

Insufficient user friendliness and lack of integration into the workflow are key impediments to the uptake of IT-based AMS interventions. Therefore, we will evaluate the physicians' experiences with the use of the app. After each introduction period all physicians of intervention wards will receive a questionnaire to collect information on experiences using the app. The questionnaire will be based on information from the literature (15), see Appendix 2.

Blinding during evaluation of outcome parameters

Given the nature of the intervention and the primary outcome assessed it will be difficult to blind the outcome assessor. To assure that assessment bias is limited we will have a subset of outcomes (at least 10%) assessed by a blinded assessor.

Statistical methods

Baseline characteristics of patients (e.g. gender, age, allergies, co-morbidities), use of antimicrobial drugs, appropriate antimicrobial therapy, and other outcome parameters will be described by entity.

For peer review only

To mitigate possible differences in time trends and baseline characteristics at patient and center level, primary analysis will be by center and effect estimates will be combined over centers using meta-analytic methods. These primary analyses will employ multivariable multilevel logistic models with time periods as fixed effects to adjust for time trend and treatment as fixed effect to estimate the odds ratio of treatment. Entities (ward/specialty) will be included as a random effect to account for the correlation of patients within entities, assuming this correlation is independent of time period of including the patient. Moreover, covariates will be included to adjust for age, gender, hospital, and specialty (medical, surgical). Similar models but using (generalized) linear multilevel models will be used for continuous outcomes or incidence/rate outcomes.

Sensitivity analyses may include the following: adjusting for additional confounders if imbalance at baseline is present; investigating deviations from the assumption that the correlation of patients within entities is independent of time period of including the patient; estimating differences in percentage (instead of odds ratios) using a (generalized) linear multilevel models with identity link and binomial error distribution; incorporating the three centers in one model. Subgroup analysis for specialty and other factors as appropriate will be conducted.

Sample size

By including 24 entities (wards/specialties) with 7 measurement periods (including one baseline period) in which the primary outcome (appropriate empiric antimicrobial drug use) will be measured in 15 patients per entity and an assumed intra-entity correlation coefficient (ICC) of 0.1, the study has a power of 81% to detect an absolute improvement in appropriate antimicrobial use by 10 percentage points from of 60% to 70%.(16) To account for potential drop-outs or a lower inclusion rate, we aim to include 36 clusters in the three participating hospitals.

Ethics and dissemination

This protocol will be initiated following approval by the institutional review board (IRB) of each participating center. Physicians will be informed about the app without obligation to use it, furthermore analysis of usage will be anonymous, therefore a waiver for informed consent for physicians has been asked. To monitor patient data, consent will be asked according to the country legislation and IRB mandate.

Dissemination

For peer review only

Results will be disseminated to healthcare professionals and researchers via presentations at national and international conferences and publications in peer-reviewed journals. Furthermore, we plan to have a workshop with interested healthcare workers after the end of the study to disseminate experiences and findings. The public will be informed through press releases. General project data and metadata will be included in the supplementary data of published articles if needed, and/or shared through university repositories.

Discussion and implications

To date, few IT-based AMS interventions have been evaluated in randomized clinical trials.(4, 10, 11) Clinical decision making by means of clinical decision support systems (CDSS) integrated in the EHR present the "gold standard" of IT-based antimicrobial stewardship. However their widespread use has been limited by some major obstacles: [1] EHRs are still not universally implemented in all hospitals in high-income countries, and even less so in low- and middle income countries [2] even if an EHR is implemented it usually lacks essential components for CDSS such as a computerized physician order entry system [3] EHRs - especially those of commercial vendors – have designs that are difficult to modify/ adapt. By developing an "app" as a software program running on smartphones and other mobile devices such as tablets, an intermediate or complementary step to full CDSS may be offered. Spectrum is a smartphone-based digital stewardship application that is customizable to local guidelines by local AMS teams. In our stepped-wedge cluster randomized trial we will evaluate the impact of this app on appropriate antimicrobial prescribing in high income countries. However, smartphone adoption is consistently strong in middle income countries such as in the Caribbean and reached low-income countries in Africa.(17, 18) More and more healthcare workers have their own electronic devices (smartphone or tablets).(7) AMS smartphone-based interventions can be made easily available, in high, low and middle income countries. The ability of this app will make antibiotic prescription guidelines more accessible for physicians all over the world, thereby helping to fight AMR.

For peer review only

References

1. van den Bosch CM, Hulscher ME, Natsch S, Wille J, Prins JM, Geerlings SE. Applicability of generic quality indicators for appropriate antibiotic use in daily hospital practice: a cross-sectional point-prevalence multicenter study. Clin Microbiol Infect. 2016;22(10):888 e1-e9.
2. Yong MK, Buising KL, Cheng AC, Thursky KA. Improved susceptibility of Gram-negative bacteria in an intensive care unit following implementation of a computerized antibiotic decision support system. J Antimicrob Chemother. 2010;65(5):1062-9.
3. McGregor JC, Weekes E, Forrest GN, Standiford HC, Perencevich EN, Furuno JP, et al. Impact of a computerized clinical decision support system on reducing inappropriate antimicrobial use: a randomized controlled trial. J Am Med Inform Assoc. 2006;13(4):378-84.
4. Baysari MT, Lehnbom EC, Li L, Hargreaves A, Day RO, Westbrook JI. The effectiveness of information technology to improve antimicrobial prescribing in hospitals: A systematic review and meta-analysis. Int J Med Inform. 2016;92:15-34.
5. Bell BG, Schellevis F, Stobberingh E, Goossens H, Pringle M. A systematic review and meta-analysis of the effects of antibiotic consumption on antibiotic resistance. BMC Infect Dis. 2014;14:13.
6. Davey P, Brown E, Charani E, Fenelon L, Gould IM, Holmes A, et al. Interventions to improve antibiotic prescribing practices for hospital inpatients. Cochrane Database Syst Rev. 2013(4):CD003543.
7. Patel RK, Sayers AE, Patrick NL, Hughes K, Armitage J, Hunter IA. A UK perspective on smartphone use amongst doctors within the surgical profession. Ann Med Surg (Lond). 2015;4(2):107-12.
8. Stergiannis P, Intas G, Toulia G, Tsolakoglou I, Kostagiolas P, Christodoulou E, et al. Clinical Use of Smartphones Among Medical and Nursing Staff in Greece: A Survey. Comput Inform Nurs. 2017;35(9):483-8.
9. Haffey F, Brady RR, Maxwell S. Smartphone apps to support hospital prescribing and pharmacology education: a review of current provision. Br J Clin Pharmacol. 2014;77(1):31-8.
10. Cresswell K, Mozaffar H, Shah S, Sheikh A. Approaches to promoting the appropriate use of antibiotics through hospital electronic prescribing systems: a scoping review. Int J Pharm Pract. 2017;25(1):5-17.
11. Rawson TM, Moore LSP, Hernandez B, Charani E, Castro-Sanchez E, Herrero P, et al. A systematic review of clinical decision support systems for antimicrobial management: are we failing to investigate these interventions appropriately? Clin Microbiol Infect. 2017;23(8):524-32.
12. Palmay L, Elligsen M, Walker SA, Pinto R, Walker S, Einarson T, et al. Hospital-wide rollout of antimicrobial stewardship: a stepped-wedge randomized trial. Clin Infect Dis. 2014;59(6):867-74.
13. Brown CA, Lilford RJ. The stepped wedge trial design: a systematic review. BMC Med Res Methodol. 2006;6:54.
14. OpenClinica LLC and collaborators. OpenClinica open source software, version 3.1.
15. Flottorp SA, Oxman AD, Krause J, Musila NR, Wensing M, Godycki-Cwirko M, et al. A checklist for identifying determinants of practice: a systematic review and synthesis of frameworks and taxonomies of factors that prevent or enable improvements in healthcare professional practice. Implement Sci. 2013;8:35.
16. Hussey MA, Hughes JP. Design and analysis of stepped wedge cluster randomized trials. Contemp Clin Trials. 2007;28(2):182-91.
17. GSMA. The Mobile Economy Latin America and the Caribbean 2018. 2018.
18. GSMA. The Mobile Economy Sub-Saharan Africa 2019. 2019.

For peer review only

19. World Health Organization. WHO Model Lists of Essential Medicines - EML – 20th edition. 2017.

For peer review only

Authors' contributions

A draft protocol was written by A. Verbon, B. Huttner, T. Tängdén, J. Conly and J.W. Mouton. During extensive calls and meetings the protocol was further shaped and fine-tuned by all authors.

Funding statement

This work is supported by Joint Programming Initiative on Antimicrobial Resistance (JPIAMR), grant number JPIAMR2017-045.

Competing interests statement

None declared.

For peer review only

Table 1: characteristics of the participating centers

**Name**	**Geneva University Hospitals**	**Erasmus MC Rotterdam**	**Uppsala University Hospital**
**Website**	www.hug-ge.ch/en/	https://www.erasmusmc.nl/	https://www.akademiska.se/en/
**Abbreviation**	HUG	EMC	UUH
**City**	Geneva	Rotterdam	Uppsala
**Country**	Switzerland	The Netherlands	Sweden
**Care level**	Primary and Tertiary Care	Tertiary Care	Tertiary Care
**Academic affiliation**	Yes	Yes	Yes
**Number of beds**	About 1’900	900	About 1´000
**Availability of antibiotic guidelines**	Paper format and PDF<br>Updated every 2 years<br>Integrated into the EHR in some units in the context of a different study (Trial registration number: NCT03120975); COMPASS units	Website containing local guidelines. Updated every 2 years. Guidelines are based on the national guidelines of SWAB	Local guidelines in paper format and online (pdf). Updated every 2-3 years. National guidelines provided by STRAMA available online and in app format, not routinely used at UUH.

For peer review only

	not participating in this study		
**Current standard of care antimicrobial stewardship**	• On demand ID consultations<br>• Review of all positive blood cultures<br>• Daily rounds on some units<br>• Approval required for certain antibiotics	• On demand ID consultations<br>• Daily rounds on Intensive care units, weekly rounds on all units<br>• Review and feedback for certain antibiotics<br>• Approval required for certain antibiotics<br>• Review of all positive blood cultures	• On demand ID consultations<br>• Daily rounds on intensive care units<br>• Adapted information and feedback to physicians at major departments 1-2 times per year

For peer review only

Table 2: PICOT of the study question

Population	Physicians involved in antimicrobial prescribing decisions for hospitalized adult patients in the participating centers
Intervention	Making a smartphone application with antimicrobial treatment recommendations available to physicians mentioned above
Comparator	Standard-of-care antimicrobial stewardship
Outcome	Appropriateness of empiric antimicrobial prescribing based on predefined criteria
Time	12 months consisting of a 6 months introduction period with 6 months follow-up to assess sustainability

For peer review only

Table 3. Secondary study parameters/endpoints and other study parameters

Outcome type	Examples	Data source
**Quantitative antimicrobial use**	• Total prescription of antimicrobial drugs1 in DDD/admission, DDD/PD, DOT/PD and DOT/admission<br>• Total prescription of broad spectrum and restricted antimicrobial drugs in DDD/admission<br>• Total prescription of antimicrobial drugs per AWaRE category(19) in DDD/admission<br>• Antimicrobial costs	• EHR<br>• Administrative data
**Patient Related**	• Length of hospital stay<br>• In hospital mortality within 30 days after admission (all cause)<br>• Unplanned hospital readmissions within 30 days after discharge<br>• Transfer to intermediate care or ICU within 30 days after admission	• EHR<br>• Administrative data
**Microbiologic and HAI**	• Incidence of healthcare facility onset *Clostridium difficile*<br>• Incidence clinical cultures with multi-drug resistant organisms (MRSA, ESBL-E, CPE, VRE, multidrug resistant *P. aeruginosa*) denominated per 1000 PD and admission	• EHR<br>• Microbiology database<br>• Infection control surveillance data

For peer review only

**Physician related**	• Uptake of the AB-assistant (total users and number of sessions per user, time spent per session, time spent per screen, number of times each screen is viewed)<br>• Actual use of app and experiences while using it	• Content Management System<br>• Survey
**Other outcomes**	• Number of infectious diseases consultations	• EHR

DDD: defined daily dose **EHR:** electronic health record; **PD:** patient- days; **HAI**: hospital acquired infections; **CMS:** content management system'; **ICU:** intensive care unit; **MRSA:** methicillin resistant staphylococcus aureus; **ESBL-E:** extended-spectrum beta-lactamase-producing E; **CPE:** carbapenemase-producing enterobacteriaceae; **CRE:** carbapenem-resistant enterobacteriaceae

1 Antimicrobials belonging to Anatomical Therapeutic Chemical Classification System class J01, J02, J03, J04, J05 (excluding anti-HIV drugs), P01, P02 and P03, oral vancomycin (A07AA09) and fidaxomicin (A07AA12).

For peer review only

Baseline No intervention	0-4 weeks	4-8 weeks	8-12 weeks	12-16 weeks	16-20 weeks	20-24 weeks	6-12 months All wards using app
All hospital wards							
Ward 31-36							
Ward 25-30							
Ward 19-24							
Ward 13-18							
Ward 7-12							
Ward 1-6							

In all time periods (total of 7) there is an uptake period of 2 weeks and a measure period of 2 weeks. During the baseline period only a measure period of 2 weeks will be performed

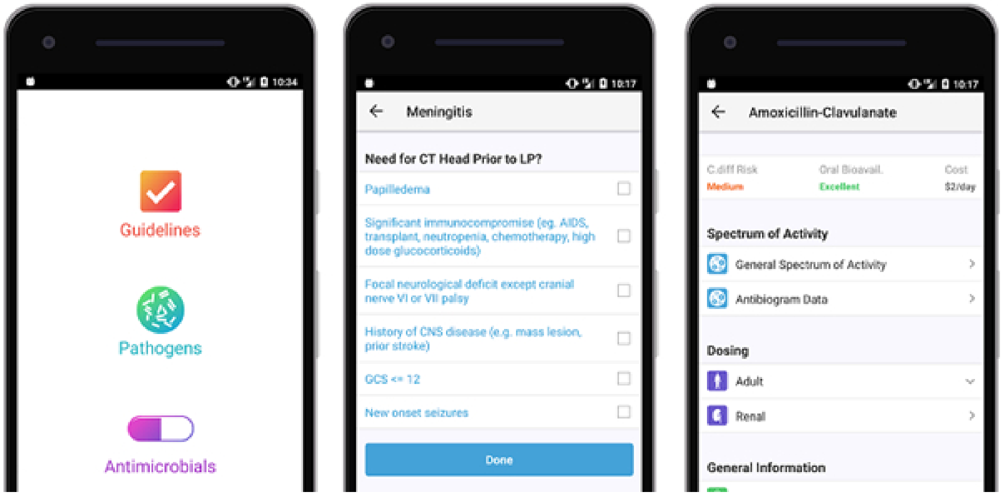

Interface of the AB-assistant app. On the left the Home screen is shown. In the middle checkboxes are shown to guide the user to the correct therapy for Meningitis. The right screen shows details about Amoxicillin-Clavulanate regarding dosing and general spectrum of activity

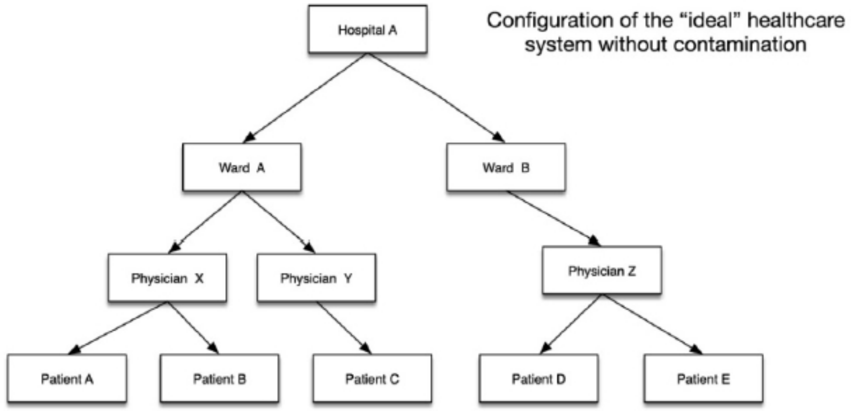
Configuration of the “ideal” healthcare system without contamination
Hospital A
Ward A
Ward B
Physician X
Physician Y
Physician Z
Patient A
Patient B
Patient C
Patient D
Patient E

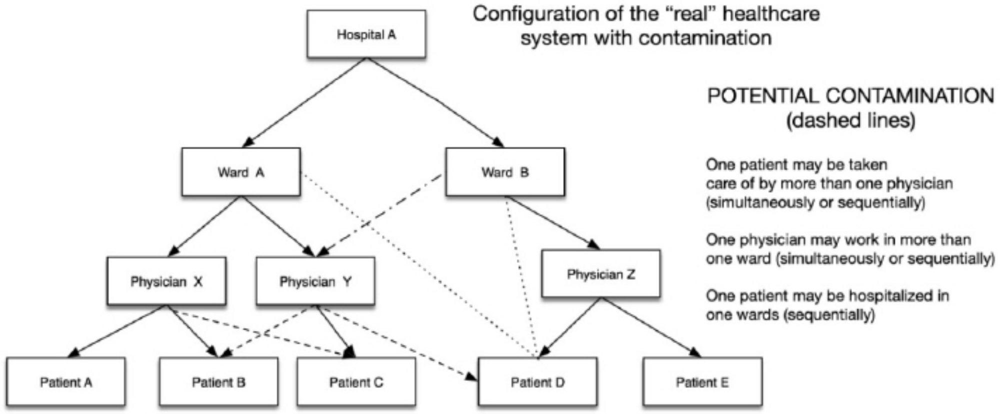
Configuration of the “real” healthcare system with contamination
Hospital A
Ward A
Ward B
Physician X
Physician Y
Physician Z
Patient A
Patient B
Patient C
Patient D
Patient E
POTENTIAL CONTAMINATION (dashed lines)
One patient may be taken care of by more than one physician (simultaneously or sequentially)
One physician may work in more than one ward (simultaneously or sequentially)
One patient may be hospitalized in one wards (sequentially)

Appendix 1

CRF for appropriate antimicrobial drug use

1. Age of patient on date on study inclusion?
2. Gender of patient?
3. Allergy listed / specified?
    a. Listed & specified
    b. Listed without specification
    c. Not listed
    d. Not assessable
4. Date of hospital admission?
5. To which ward is patient admitted?
6. Date of admission to this ward unit?
7. Date of discharge from this ward/unit?
8. Indication listed or documented in admission note within 24 hours after starting antimicrobial drugs?

9.1.1 On which ward was the first antimicrobial drug prescribed?

9.1.2 What antimicrobial drug was prescribed?

9.1.3 On what date was the antimicrobial drug prescribed?

9.1.4 At what time was the antimicrobial drug prescribed?

9.1.5 Initial route of first antimicrobial drug prescribed?

9.1.6 Were there more than one therapeutic antimicrobial drug prescribed?

10. Correct choice of antimicrobial drugs according to the guidelines at time of prescribing according to indication?
    a. Fully concordant
    b. Partially concordant
    c. Not concordant
    d. No guideline available for specific indication
    e. Not assessable (no indication written)
11. Dosing regimen correct for all of the given antimicrobial drugs (adjusted for renal function too)?
12. Initial route of administration correct according to local guideline of all antimicrobial drugs?
13. Blood cultures performed?
14. Cultures or relevant microbial testing from suspected sites of infection performed according to local guideline (excluding blood cultures)?

For peer review only

15. PCR/Culture taken during this admission positive for C. difficile or MDRO (MRSA, ESBL-E, CPE, VRE, multidrug resistant P. aeruginosa)?
16. De-escalation from empirical to pathogen-directed therapy within 5 days after starting treatment for all antimicrobial drugs?
17. Switch IV to PO after 48-96h if clinically appropriate according to local guidelines?
18. Duration of antibiotic plan documented in medical chart? (between admission to this ward and day 5)
19. Duration of actual given antimicrobial drugs in accordance to local guidelines?
20. Therapeutic drug monitoring (TDM) according to local guidelines (aminoglycosides, glycopeptides, azoles) for all antimicrobial drugs?
21. Did the patient receive antimicrobial drugs <30 days before start of (empirical/targeted) treatment?
22. Have any of the following complications occurred?
    a. In-hospital mortality within 30 days after hospital admission (all cause)
    b. Transfer to intermediate care
    c. Transfer to ICU
    d. Unplanned readmission within 30 days after this admission
    e. Combination of answers above
    f. No
23. Is there an ID specialist / clinical microbiologist consulted for this treatment episode?
    a. Yes, in the first 5 days after start of antimicrobial treatment
    b. Yes, after 5 days of start antimicrobial treatment
    c. No
    d. Not assessable

For peer review only

Appendix 2: Post-intervention questionnaire

For each of the questions below, circle the response that characterizes best how you feel about each statement,

where: 1 = Strongly disagree, 2 = Disagree, 3 = Neither disagree nor agree, 4 = Agree, 5 = Strongly agree

	Strongly disagree	Disagree	Neither agree nor disagree	Agree	Strongly agree
1. This app explains clearly what I have to do and in which order	1	2	3	4	5
2. This app includes every step of appropriate antibiotic use in the hospital	1	2	3	4	5
3. This app is too complex for use in daily practice	1	2	3	4	5
4. Guidelines in the app were accessible	1	2	3	4	5
5. Guidelines in the app were consistent with other guidelines	1	2	3	4	5
6. This app fits in current practices	1	2	3	4	5
7. The benefits of using the app are clear	1	2	3	4	5
8. This app is feasible for all my patients who receive IV antibiotics	1	2	3	4	5
9. This app is a threat to my professional autonomy	1	2	3	4	5

For peer review only

10. I already knew the guidelines in the app	1	2	3	4	5
11. I trust the guidelines in the app	1	2	3	4	5

	Strongly disagree	Disagree	Neither agree nor disagree	Agree	Strongly agree
12. I agree with the guidelines in the app	1	2	3	4	5
13. I have a positive attitude towards guidelines in general	1	2	3	4	5
14. I expect that this app will improve the quality of my antibiotic prescriptions	1	2	3	4	5
15. I could not observe benefits of using the app	1	2	3	4	5
16. It is part of my job to use this app	1	2	3	4	5
17. I intend to use the app to assess the guidelines	1	2	3	4	5
18. I am capable of using this app	1	2	3	4	5
19. It does not take a lot of effort to use this app	1	2	3	4	5
20. I was able to try the app before using it for actual prescriptions	1	2	3	4	5
21. I have enough knowledge and expertise to use the app adequately	1	2	3	4	5

For peer review only

22. I have good previous experiences with working with an app	1	2	3	4	5
23. Colleagues will support me to use this	1	2	3	4	5
24. Supervisors will support me to use this app	1	2	3	4	5
	Strongly disagree	Disagree	Neither agree nor disagree	Agree	Strongly agree
25. Nurses will support me to use this app	1	2	3	4	5
26. Colleagues will use this app	1	2	3	4	5
27. Colleagues will expect me to use the app	1	2	3	4	5
28. Supervisors will expect me to use the app	1	2	3	4	5
29. Nurses will expect me to use the app	1	2	3	4	5
30. Patient will be satisfied that this app is being used	1	2	3	4	5
31. I expect that this app will improve the patient's antibiotic treatment	1	2	3	4	5
32. There are enough financial resources to use the app as it is meant to be used	1	2	3	4	5
33. I can use the app while working with the electronic health records	1	2	3	4	5

For peer review only

system					
34. External support is needed to implement the app on organizational level	1	2	3	4	5

For peer review only

BMJ Open

Study protocol for an international, multicenter stepped-wedge cluster randomized trial to evaluate the impact of a digital antimicrobial stewardship smartphone application

Journal:	*BMJ Open*
Manuscript ID	bmjopen-2019-033640.R1
Article Type:	Protocol
Date Submitted by the Author:	23-Dec-2019
Complete List of Authors:	Helou, R.I.; Erasmus Medical Center, Department of Medical Microbiology and Infectious Diseases<br>Catho, Gaud ; Hopitaux Universitaires de Geneve, Department of Infectious Diseases<br>Peyravi Latif, Annabel; Uppsala University Hospital, Department of Infectious Diseases<br>Mouton, Johan; Erasmus Medical Center, Department of Medical Microbiology and Infectious Diseases<br>Hulscher, M; Radboud University Medical Center, Scientific Center for Quality of Healthcare (IQ healthcare), Radboud Institute for Health Sciences<br>Teerenstra, Steven; Radboud University Medical Center, Department for Health Evidence, Radboud Institute for Health Sciences<br>Conly, John; University of Calgary and Alberta Health Services, Department of Medicine<br>Huttner, Benedikt D; Hopitaux Universitaires de Geneve, Department of Infectious Diseases<br>Tängdén, Thomas; Uppsala University Hospital, Department of Infectious Diseases<br>Verbon, Annelies ; Erasmus Medical Center, Department of Medical Microbiology and Infectious Diseases
**Primary Subject Heading**:	Infectious diseases
Secondary Subject Heading:	Health informatics
Keywords:	INFECTIOUS DISEASES, MICROBIOLOGY, Protocols & guidelines < HEALTH SERVICES ADMINISTRATION & MANAGEMENT, Information technology < BIOTECHNOLOGY & BIOINFORMATICS, THERAPEUTICS

SCHOLARONE™
Manuscripts

I, the Submitting Author has the right to grant and does grant on behalf of all authors of the Work (as defined in the below author licence), an exclusive licence and/or a non-exclusive licence for contributions from authors who are: i) UK Crown employees; ii) where BMJ has agreed a CC-BY licence shall apply, and/or iii) in accordance with the terms applicable for US Federal Government officers or employees acting as part of their official duties; on a worldwide, perpetual, irrevocable, royalty-free basis to BMJ Publishing Group Ltd ("BMJ") its licensees and where the relevant Journal is co-owned by BMJ to the co-owners of the Journal, to publish the Work in this journal and any other BMJ products and to exploit all rights, as set out in our licence.

The Submitting Author accepts and understands that any supply made under these terms is made by BMJ to the Submitting Author unless you are acting as an employee on behalf of your employer or a postgraduate student of an affiliated institution which is paying any applicable article publishing charge ("APC") for Open Access articles. Where the Submitting Author wishes to make the Work available on an Open Access basis (and intends to pay the relevant APC), the terms of reuse of such Open Access shall be governed by a Creative Commons licence – details of these licences and which Creative Commons licence will apply to this Work are set out in our licence referred to above.

Other than as permitted in any relevant BMJ Author's Self Archiving Policies, I confirm this Work has not been accepted for publication elsewhere, is not being considered for publication elsewhere and does not duplicate material already published. I confirm all authors consent to publication of this Work and authorise the granting of this licence.

For peer review only

Study protocol for an international, multicenter stepped-wedge cluster randomized trial to evaluate the impact of a digital antimicrobial stewardship smartphone application

For peer review only

R.I. Helou[1], G. Catho[2], A. Peyravi Latif[3], J.W. Mouton[1], M.E.J.L. Hulscher[4], S. Teerenstra[5], J. Conly[6], B. D. Huttner[2], T. Tängdén[3], A. Verbon[1*]

1) Department of Medical Microbiology and Infectious Diseases, Erasmus Medical Center, Rotterdam, The Netherlands
2) Department of Infectious Diseases, Geneva University Hospitals and Faculty of Medicine, Geneva, Switzerland
3) Department of Infectious Diseases, Uppsala University Hospital, Uppsala, Sweden
4) Scientific Center for Quality of Healthcare (IQ healthcare), Radboud Institute for Health Sciences, Radboud University Medical Center
5) Department for Health Evidence, Radboud Institute for Health Sciences, Radboud University Medical Center
6) Department of Medicine, University of Calgary and Alberta Health Services, Calgary, Alberta, Canada

*) corresponding author
email: a.verbon@erasmusmc.nl

Abstract

Introduction With the widespread use of electronic health records (EHR) and handheld electronic devices in hospitals, informatics-based antimicrobial stewardship (AMS) interventions hold great promise as tools to promote appropriate antimicrobial drug prescribing. However, more research is needed to evaluate their optimal design and impact on quantity and quality of antimicrobial prescribing.

Methods and analysis Use of smartphone-based digital stewardship applications (app) with local guideline directed empirical antimicrobial use by physicians will be compared to antimicrobial prescription as per usual as primary outcome in three hospitals in the Netherlands, Sweden and Switzerland. Secondary outcomes will include antimicrobial use metrics, clinical and process outcomes. A multicenter stepped-wedge cluster randomized trial will randomize entities defined as wards or specialty regarding time of introduction of the intervention. We will include 36 hospital entities with 7 measurement periods in which the primary outcome will be measured in 15 participating patients per time period per cluster. At participating wards, patients of at least 18 years of age using antimicrobials will be included. After a baseline period of 2-week measurements, 6 periods of 4 weeks will follow in which the intervention is introduced in 6 wards (in 3 hospitals) until all 36 wards have implemented the intervention. Thereafter, we allow use of the app by everyone, and evaluate the sustainability of the app use 6 months later.

Ethics and dissemination This protocol will be initiated following approval by the institutional review board (IRB) of each participating center. Results will be disseminated via media, to healthcare professionals via professional training and meetings and to researchers via conferences and publications.

Registration details This study is registered on ClinicalTrials.gov under identifier NCT03793946.

For peer review only

Strengths and limitations of this study

- This study will be an international randomized, multi-center, clinical trial using a rigorous design and methodology to evaluate the impact of an AMS smartphone app for the hospital setting
- The app can easily be adapted to local guidelines which is a key feature for potential future use in other settings
- The primary outcome is appropriate empiric antimicrobial therapy. The study is conducted in three hospitals with relatively high rates of appropriate antibiotic use, which may theoretically lead to a higher risk of a negative trial
- In this cluster randomized trial, wards/specialties are the unit of randomization and there is a risk of contamination through physicians changing between wards or among specialties, although this risk is lower than for a single-center randomized trial. Restricted access to the software and tracking of app use will be applied to monitor possible contamination.

Introduction

Antimicrobial drugs are an indispensable part of modern medicine, which depends on effective drugs for prophylaxis and treatment. Yet, only around 40-70% of empiric antimicrobial regimens are considered appropriate with regard to their indication, the choice of agent, dosing or duration.(1-4) Inappropriate antimicrobial use enhances the risks of treatment failure and side effects in the individual patient, and accelerates selection and transmission of antimicrobial-resistant pathogens in health care settings and the community.(5) Many antimicrobial stewardship (AMS) interventions have been shown to increase appropriateness of antimicrobial drug prescribing.(6)

A software application (app), which is compatible with mobile electronic devices such as smartphones and tablets, and increases the availability of existing national and local antibiotic guidelines plus local antibiograms, may improve antimicrobial prescribing, reduce medication errors and ultimately improve patient outcome. Due to its highly customizable nature, a digital app has the potential to become an effective AMS tool in all countries.

Mobile-software apps seem particularly interesting for the purpose of AMS, since they do not require access to electronic health records or computerized physician order entry systems, that are costly, difficult to maintain and not easily customizable. Most healthcare workers own a smartphone and often use it to access information to assist in their treatment decisions.(7, 8) The

For peer review only

vast majority of available apps developed to support prescription of medicines are limited to knowledge content and do not provide decision support algorithms. Further, they have rarely been evaluated in clinical trials.(9) To date, most reported digital AMS interventions are single-center studies with limited internal validity due to methodological limitations and uncertain external validity.(4, 10, 11)

In the present study, we will evaluate the implementation and impact of a smartphone-based software application (the AB-Assistant, Spectrum, Calgary, Canada) adapted to local conditions in an international clinical trial using a stepped-wedge cluster randomized design and appropriateness of empiric antimicrobial prescribing as the primary outcome.

Methods and analysis

Setting

This study will be conducted in three academic, tertiary care hospitals in Europe: Erasmus University Medical Center, Rotterdam, the Netherlands; Uppsala University Hospital, Uppsala, Sweden; and Geneva University Hospitals, Geneva, Switzerland (Table 1).

Study design

We plan to conduct an international, multicenter, stepped wedge cluster randomized trial to assess whether making a smartphone application for AMS (Spectrum) can improve the appropriateness of antimicrobial prescribing in hospitalized patients. Table 2 illustrates the study question according to the PICOT framework.

In total, 36 medical and surgical entities (defined as wards (Sweden) or specialties (Switzerland, The Netherlands) will be included in the study using a stepwise design with 6 entities (2 entities per participating hospital) 4-weekly at 6 introduction moments (Figure 1). The primary outcome is appropriateness of empiric antimicrobial prescriptions assessed prior to implementation and up to 12 months after the intervention (after 6 months free use of the app). Empiric therapy is defined as (1) treatment started based on clinical evaluation before culture results are known, (2) the duration of treatment is longer than 24 hours (from the moment of prescription) and (3) the treatment is not specifically recorded as prophylactic. Initial targeted therapy for which there is a local guideline available (e.g. C. difficile) may be assessed for appropriateness.

For peer review only

The entities will be randomized as to the time period of making the app available (see intervention below). The order of implementation will be determined by a computer-generated list of random numbers programmed by a statistician otherwise not involved in the trial and AMS activities. Randomization will be stratified by type of specialty (medicine, surgery etc.). After a baseline period of 2-week measurements, 6 periods of 4 weeks will follow in which the intervention is introduced in 6 entities (in 3 hospitals) until all entities have implemented the intervention. We plan to include 36 entities in total (see sample size calculation below) during the 6 introduction moments and at the end of the inclusion time we will allow the use of the app by everyone, also wards/specialties not included in the study. In this way, the trend in appropriate antimicrobial use can be monitored per ward/specialty and the decrease of antimicrobial use and appropriateness of empirical antimicrobial therapy can be followed during the stepwise implementation.(12) At 12 months (after 6 months free use of the app) we will have a 2-week measurement period to evaluate the sustainability of the intervention.

Justification of the stepped wedge cluster randomized study design

The stepped-wedge cluster randomized design was chosen after considering several advantages and disadvantages of randomization at the patient, physician, specialty and ward level. Patients are the “classic unit” of randomization and outcomes are often assessed at the patient level. However, there is a large risk of contamination of the intervention since one patient is often taken care of by many different physicians (see Figure 2). Furthermore, the study intervention targets physicians and not patients, therefore precluding randomization at the patient level as an option. Randomization at the physician-level is, however, equally problematic since prescribing decisions in hospitals are often made by teams of physicians, making it difficult to assign a decision to a specific individual physician and introducing the risk of contamination between physicians within a team. We therefore decided to use the wards/specialty as entity of randomization since it minimizes the risk of contamination and reflects “real-life” antimicrobial stewardship in hospitals where interventions are usually implemented at a ward/specialty level.

The stepped-wedge design, which involves the randomized and sequential rollout of the intervention to clusters over time, was chosen because it can model and adjust for underlying temporal trends and results in all clusters having access to the intervention at the end of the rollout period which seems desirable from an antimicrobial stewardship perspective.(13)

For peer review only

Study population

In our study, the intervention will be targeted at physicians that prescribe antimicrobials. To avoid possible contamination, the prescribers will be randomized at the ward/specialty level and not individually (see above). Process parameters will be determined in physicians who have been randomized to use the app per the process indicated above. Outcome parameters such as appropriate antimicrobial use will be determined at the patient level at participating wards (control and intervention) who meet the following criteria: being a medical or surgical specialty patient at least 18 years of age for whom antimicrobial drugs are prescribed.

Intervention

The Spectrum application, available for iOS and Android and on the web, has been developed in Canada (www.spectrum.app) and is now being used in over 50 hospitals, mainly in Canada and the United States of America. The app has been developed with the help of human factor experts and integrates multiple resources into a unified user-friendly interface for use at point-of-care to improve antimicrobial prescribing (Figure 3).

For the purpose of this study the app and the content management system were extended to also support languages other than English (French, Dutch, Swedish) and will be used for prescribing guidelines, pathogens, antimicrobial drugs and antibiograms. App content such as local guidelines, information regarding antimicrobial drugs, and micro-organisms of each hospital was entered by investigators of the Dutch, Swiss and Swedish hospitals (RH, GC, BH, APL). App content is identical to content of the local guidelines if these are published on a website. Minor discrepancies may occur if the local guidelines are published in a digital document (i.e. PDF) or on paper. The app can be more up to date in those cases.

The allocation of the timing to introduce the app will be concealed to the participating cluster up until 2 weeks before initiating use of the intervention. At the start of the intervention physicians on the intervention ward(s) will be informed about the function and content of the app. During the trial, only physicians on the intervention wards will be whitelisted to be able to use the app. Access to the app will be centrally withdrawn if a physician leaves the intervention ward (for example to work on a different (i.e. non-intervention) ward). The conventional route of guidelines (e.g. portable document formats (PDF), website or printed booklet) will still be available at all times. Physicians can freely choose between the app and the conventional route. Physicians on control wards will only be able to access the guidelines using the conventional route.

For peer review only

Patient and Public Involvement

No patients or public have been included in the study design.

Outcome parameters

Outcome parameters (Table 3) will be monitored using a digital CRF (Appendix 1) in the software application OpenClinica. (14)

Primary outcome parameter

Appropriateness of empiric antimicrobial therapy expressed as number and percentage of appropriate treatment episodes compared to all assessed treatment episodes.

Definitions for the primary outcome are:

- antimicrobial drugs assessed are ATC classes J01, J02, J03, J04, J05 (excluding anti-HIV drugs), P01, P02 and P03.
- treatment episodes are defined as the duration of treatment given for a specific infectious episode with gaps of treatment of no longer than one day.
- treatments are antimicrobials started during the measurement period at the participating entity. If a treatment was started within 24 hours before transfer in the emergency room or other ward and has not been changed during the first 24 hour stay at the ward, the treatment will be considered in accordance with the choice of the ward physician.
- treatments are in patients either newly admitted to the participating entities or already hospitalized during the pre-intervention period.
- appropriateness of empiric therapy includes choice, route and the dose of the antimicrobial drugs.
- appropriateness of therapy is in concordance with local treatment guidelines. If no guidelines are available, or if the treatment deviates from the guidelines or if treatment is guideline concordant but treatment should have deviated from the guideline (e.g. because of allergies, colonization with multidrug-resistant bacteria, other microbiological results, interactions, comorbidities or recent exposure to antimicrobials), anonymized cases will be discussed among 2 independent local infectious disease (ID) physicians. If they do not agree a third independent local ID physician will be consulted to adjudicate.
- for each patient only the first treatment episode during the measurement period will be evaluated. For patients treated for >1 infection (e.g. pneumonia and *Clostridium difficile* infection) appropriateness will be assessed for all infections combined (e.g. if

For peer review only

treatment is appropriate for *C. difficile* infection but inappropriate for pneumonia the treatment would be categorized as inappropriate).

Secondary outcome parameters

- Total prescription of antimicrobial drugs on the cluster-level expressed as DDD and DOT denominated by patient-days and admission; subcategories by type of antimicrobial (antibiotic, antifungal, etc.), WHO AWaRE categories and restricted antimicrobial drugs (Table 3).
- Clinical secondary outcomes: length of hospital stay, in hospital mortality and readmissions within 30 days after discharge, incidence/prevalence of key antimicrobial resistant (AMR) pathogens such as *C. difficile*.
- Process parameters of the intervention: penetration of the AB-assistant (total users and number of use of app).

Alongside the RCT, an uptake analysis will be performed to assess actual use of the app as well as an evaluation of use.

Uptake analysis

A number of parameters regarding use of the app will be collected and made available on a secure dashboard to authorized users at each site. These include number of monthly users and monthly sessions, number of times each antimicrobial, pathogen or clinical pathway screen is viewed, use by hospital, department, and medical specialism. These data will enable in-app behavior to be correlated with actual prescribing behavior on ward level and site-wide basis, thereby strengthening the association of the intervention to observed outcomes.

Evaluation of use

Insufficient user friendliness and lack of integration into the workflow are key impediments to the uptake of IT-based AMS interventions. Therefore, we will evaluate the physicians' experiences with the use of the app. After each introduction period all physicians of intervention wards will receive a questionnaire to collect information on experiences using the app. The questionnaire will be based on information from the literature (15), see Appendix 2.

Blinding during evaluation of outcome parameters

For peer review only

Given the nature of the intervention and the primary outcome assessed it will be difficult to blind the outcome assessor. To assure that assessment bias is limited we will have a subset of outcomes (10%) assessed by an independent investigator blinded to the study period.

Statistical methods

Baseline characteristics of patients (e.g. gender, age, allergies, co-morbidities), use of antimicrobial drugs, appropriate antimicrobial therapy, and other outcome parameters will be described by entity.

To mitigate possible differences in time trends and baseline characteristics at patient and center level, primary analysis will be by center and effect estimates will be combined over centers using meta-analytic methods. These primary analyses will employ multivariable multilevel logistic models with time periods as fixed effects to adjust for time trend and treatment as fixed effect to estimate the odds ratio of treatment. Entities (ward/specialty) will be included as a random effect to account for the correlation of patients within entities, assuming this correlation is independent of time period of including the patient. Moreover, covariates will be included to adjust for age, gender, hospital, and specialty (medical, surgical). Similar models but using (generalized) linear multilevel models will be used for continuous outcomes or incidence/rate outcomes.

Sensitivity analyses may include the following: adjusting for additional confounders if imbalance at baseline is present; investigating deviations from the assumption that the correlation of patients within entities is independent of time period of including the patient; estimating differences in percentage (instead of odds ratios) using a (generalized) linear multilevel models with identity link and binomial error distribution; incorporating the three centers in one model.

Subgroup analysis for specialty and other factors as appropriate will be conducted.

Sample size

By including 24 entities (wards/specialties) with 7 measurement periods (including one baseline period) in which the primary outcome (appropriate empiric antimicrobial drug use) will be measured in 15 participating patients per entity per time period and an assumed intra-entity correlation coefficient (ICC) of 0.1, the study has a power of 81% to detect an absolute improvement in appropriate antimicrobial use by 10 percentage points from of 60% to 70%.(16) To account for potential drop-outs or a lower inclusion rate, we aim to include 36 clusters in the three participating hospitals.

Ethics and dissemination

For peer review only

This protocol will be initiated following approval by the institutional review board (IRB) of each participating center. Ethical approval has been obtained from the Medical Ethics Committee (METC) Erasmus MC, Rotterdam, the Netherlands, number MEC-2019-0172 and from the Commission cantonale d'éthique de la recherche (CCER), Geneva, Switzerland, number 2019-01061. Physicians will be informed about the app without obligation to use it, furthermore analysis of usage will be anonymous, therefore a waiver for informed consent for physicians has been asked. However, participating physicians will be asked for a digital informed consent the first time they use the app. To monitor patient data, consent will be asked according to the country legislation and IRB mandate.

Dissemination

Results will be disseminated to healthcare professionals and researchers via presentations at national and international conferences and publications in peer-reviewed journals. Furthermore, we plan to have a workshop with interested healthcare workers after the end of the study to disseminate experiences and findings. The public will be informed through press releases.

General project data and metadata will be included in the supplementary data of published articles if needed, and/or shared through university repositories.

Discussion and implications

To date, few IT-based AMS interventions have been evaluated in randomized clinical trials.(4, 10, 11) Clinical decision making by means of clinical decision support systems (CDSS) integrated in the EHR present the “gold standard” of IT-based antimicrobial stewardship. However their widespread use has been limited by some major obstacles: [1] EHRs are still not universally implemented in all hospitals in high-income countries, and even less so in low- and middle income countries [2] even if an EHR is implemented it usually lacks essential components for CDSS such as a computerized physician order entry system [3] EHRs - especially those of commercial vendors – have designs that are difficult to modify/ adapt. By developing an “app” as a software program running on smartphones and other mobile devices such as tablets, an intermediate or complementary step to full CDSS may be offered. Spectrum is a smartphone-based digital stewardship application that is customizable to local guidelines by local AMS teams. In our stepped-wedge cluster randomized trial we will evaluate the impact of this app on appropriate antimicrobial prescribing in high income countries. However, smartphone adoption is consistently strong in middle income countries such as in the Caribbean and reached low-income countries in Africa.(17, 18) More and more healthcare workers have their own electronic devices (smartphone

For peer review only

or tablets).(7) AMS smartphone-based interventions can be made easily available, in high, low and middle income countries. The ability of this app will make antibiotic prescription guidelines more accessible for physicians all over the world, thereby helping to fight AMR.

References

1. van den Bosch CM, Hulscher ME, Natsch S, Wille J, Prins JM, Geerlings SE. Applicability of generic quality indicators for appropriate antibiotic use in daily hospital practice: a cross-sectional point-prevalence multicenter study. Clin Microbiol Infect. 2016;22(10):888 e1-e9.
2. Yong MK, Buising KL, Cheng AC, Thursky KA. Improved susceptibility of Gram-negative bacteria in an intensive care unit following implementation of a computerized antibiotic decision support system. J Antimicrob Chemother. 2010;65(5):1062-9.
3. McGregor JC, Weekes E, Forrest GN, Standiford HC, Perencevich EN, Furuno JP, et al. Impact of a computerized clinical decision support system on reducing inappropriate antimicrobial use: a randomized controlled trial. J Am Med Inform Assoc. 2006;13(4):378-84.
4. Baysari MT, Lehnbom EC, Li L, Hargreaves A, Day RO, Westbrook JI. The effectiveness of information technology to improve antimicrobial prescribing in hospitals: A systematic review and meta-analysis. Int J Med Inform. 2016;92:15-34.
5. Bell BG, Schellevis F, Stobberingh E, Goossens H, Pringle M. A systematic review and meta-analysis of the effects of antibiotic consumption on antibiotic resistance. BMC Infect Dis. 2014;14:13.
6. Davey P, Brown E, Charani E, Fenelon L, Gould IM, Holmes A, et al. Interventions to improve antibiotic prescribing practices for hospital inpatients. Cochrane Database Syst Rev. 2013(4):CD003543.
7. Patel RK, Sayers AE, Patrick NL, Hughes K, Armitage J, Hunter IA. A UK perspective on smartphone use amongst doctors within the surgical profession. Ann Med Surg (Lond). 2015;4(2):107-12.
8. Stergiannis P, Intas G, Toulia G, Tsolakoglou I, Kostagiolas P, Christodoulou E, et al. Clinical Use of Smartphones Among Medical and Nursing Staff in Greece: A Survey. Comput Inform Nurs. 2017;35(9):483-8.
9. Haffey F, Brady RR, Maxwell S. Smartphone apps to support hospital prescribing and pharmacology education: a review of current provision. Br J Clin Pharmacol. 2014;77(1):31-8.
10. Cresswell K, Mozaffar H, Shah S, Sheikh A. Approaches to promoting the appropriate use of antibiotics through hospital electronic prescribing systems: a scoping review. Int J Pharm Pract. 2017;25(1):5-17.
11. Rawson TM, Moore LSP, Hernandez B, Charani E, Castro-Sanchez E, Herrero P, et al. A systematic review of clinical decision support systems for antimicrobial management: are we failing to investigate these interventions appropriately? Clin Microbiol Infect. 2017;23(8):524-32.

For peer review only

12. Palmay L, Elligsen M, Walker SA, Pinto R, Walker S, Einarson T, et al. Hospital-wide rollout of antimicrobial stewardship: a stepped-wedge randomized trial. Clin Infect Dis. 2014;59(6):867-74.
13. Brown CA, Lilford RJ. The stepped wedge trial design: a systematic review. BMC Med Res Methodol. 2006;6:54.
14. OpenClinica LLC and collaborators. OpenClinica open source software, version 3.1.
15. Flottorp SA, Oxman AD, Krause J, Musila NR, Wensing M, Godycki-Cwirko M, et al. A checklist for identifying determinants of practice: a systematic review and synthesis of frameworks and taxonomies of factors that prevent or enable improvements in healthcare professional practice. Implement Sci. 2013;8:35.
16. Hussey MA, Hughes JP. Design and analysis of stepped wedge cluster randomized trials. Contemp Clin Trials. 2007;28(2):182-91.
17. GSMA. The Mobile Economy Latin America and the Caribbean 2018. 2018.
18. GSMA. The Mobile Economy Sub-Saharan Africa 2019. 2019.
19. World Health Organization. WHO Model Lists of Essential Medicines - EML – 20th edition. 2017.

For peer review only

Authors' contributions

A draft protocol was written by A. Verbon, B. Huttner, T. Tängdén, J. Conly and J.W. Mouton. During extensive calls and meetings the protocol was further shaped and fine-tuned by all authors.

Funding statement

This work is supported by Joint Programming Initiative on Antimicrobial Resistance (JPIAMR), grant number JPIAMR2017-045.

Competing interests statement

None declared.

For peer review only

Table 1: characteristics of the participating centers

**Name**	**Geneva University Hospitals**	**Erasmus MC Rotterdam**	**Uppsala University Hospital**
**Website**	www.hug-ge.ch/en/	https://www.erasmusmc.nl/	https://www.akademiska.se/en/
**Abbreviation**	HUG	EMC	UUH
**City**	Geneva	Rotterdam	Uppsala
**Country**	Switzerland	The Netherlands	Sweden
**Care level**	Primary and Tertiary Care	Tertiary Care	Tertiary Care
**Academic affiliation**	Yes	Yes	Yes
**Number of beds**	About 1’900	900	About 1´000

For peer review only

**Availability of antibiotic guidelines**	Paper format and PDF<br>Updated every 2 years<br>Integrated into the EHR in some units in the context of a different study (Trial registration number: NCT03120975); COMPASS units not participating in this study	Website containing local guidelines. Updated every 2 years. Guidelines are based on the national guidelines of SWAB	Local guidelines in paper format and online (pdf). Updated every 2-3 years. National guidelines provided by STRAMA available online and in app format, not routinely used at UUH.
**Current standard of care antimicrobial stewardship**	• On demand ID consultations<br>• Review of all positive blood cultures<br>• Daily rounds on some units<br>• Approval required for certain antibiotics	• On demand ID consultations<br>• Daily rounds on Intensive care units, weekly rounds on all units<br>• Review and feedback for certain antibiotics<br>• Approval required for certain antibiotics<br>• Review of all positive blood cultures	• On demand ID consultations<br>• Daily rounds on intensive care units<br>• Adapted information and feedback to physicians at major departments 1-2 times per year

For peer review only

Table 2: PICOT of the study question

Population	Physicians involved in antimicrobial prescribing decisions for hospitalized adult patients in the participating centers
Intervention	Making a smartphone application with antimicrobial treatment recommendations available to physicians mentioned above
Comparator	Standard-of-care antimicrobial stewardship
Outcome	Appropriateness of empiric antimicrobial prescribing based on predefined criteria
Time	12 months consisting of a 6 months introduction period with 6 months follow-up to assess sustainability

For peer review only

468
469

470 **Table 3. Secondary study parameters/endpoints and other study parameters**

Outcome type	Examples	Data source
**Quantitative antimicrobial use**	• Total prescription of antimicrobial drugs1 in DDD/admission, DDD/PD, DOT/PD and DOT/admission<br>• Total prescription of broad spectrum and restricted antimicrobial drugs in DDD/admission<br>• Total prescription of antimicrobial drugs per AWaRE category(19) in DDD/admission<br>• Antimicrobial costs	• EHR<br>• Administrative data
**Patient Related**	• Length of hospital stay<br>• In hospital mortality within 30 days after admission (all cause)<br>• Unplanned hospital readmissions within 30 days after discharge<br>• Transfer to intermediate care or ICU within 30 days after admission	• EHR<br>• Administrative data
**Microbiologic and HAI**	• Incidence of healthcare facility onset *Clostridium difficile*<br>• Incidence clinical cultures with multi-drug resistant organisms (MRSA, ESBL-E, CPE, VRE, multidrug resistant *P. aeruginosa*) denominated per 1000 PD and admission	• EHR<br>• Microbiology database<br>• Infection control surveillance data

For peer review only

**Physician related**	• Uptake of the AB-assistant (total users and number of sessions per user, time spent per session, time spent per screen, number of times each screen is viewed)<br>• Actual use of app and experiences while using it	• Content Management System<br>• Survey
**Other outcomes**	• Number of infectious diseases consultations	• EHR

DDD: defined daily dose **EHR:** electronic health record; **PD:** patient- days; **HAI**: hospital acquired infections; **CMS:** content management system'; **ICU:** intensive care unit; **MRSA:** methicillin resistant staphylococcus aureus; **ESBL-E:** extended-spectrum beta-lactamase-producing E; **CPE:** carbapenemase-producing enterobacteriaceae; **CRE:** carbapenem-resistant enterobacteriaceae

1 Antimicrobials belonging to Anatomical Therapeutic Chemical Classification System class J01, J02, J03, J04, J05 (excluding anti-HIV drugs), P01, P02 and P03, oral vancomycin (A07AA09) and fidaxomicin (A07AA12).

For peer review only

Figure legends

Figure 1 - Study design

In all time periods (total of 7) there is an uptake period of 2 days and a measure period of 26 days. During the baseline period only a measure period of 2 weeks will be performed.

Figure 2 - Interface of the AB-assistant app

Interface of the AB-assistant app. On the left the Home screen is shown. In the middle checkboxes are shown to guide the user to the correct therapy for Meningitis. The right screen shows details about Amoxicillin-Clavulanate regarding dosing and general spectrum of activity.

Figure 3 - Schema of potential contamination

The potential contamination in a hospital, in and between wards, physicians and patients.

For peer review only

For peer review only - http://bmjopen.bmj.com/site/about/guidelines.xhtml

Baseline No intervention	0-4 weeks	4-8 weeks	8-12 weeks	12-16 weeks	16-20 weeks	20-24 weeks	6-12 months All wards using app
All hospital wards							
Ward 31-36							
Ward 25-30							
Ward 19-24							
Ward 13-18							
Ward 7-12							
Ward 1-6							

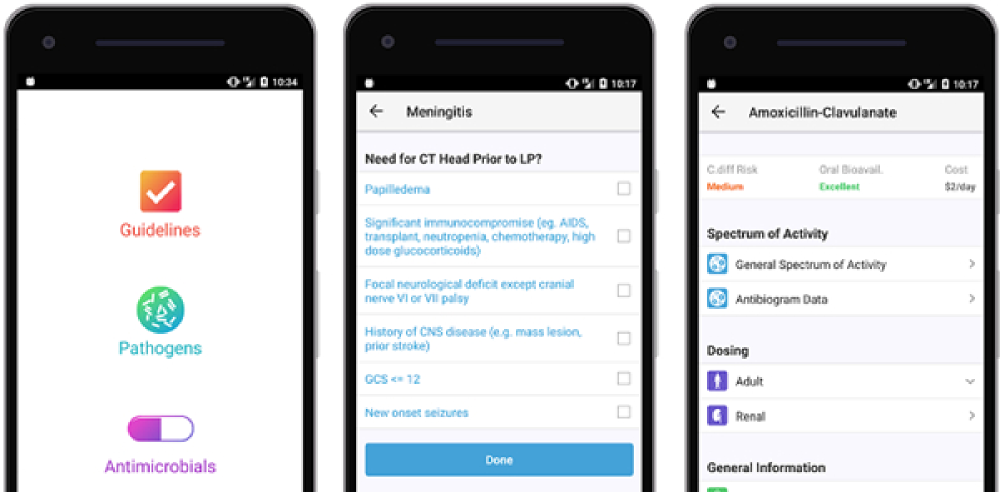
Guidelines
Pathogens
Antimicrobials
Meningitis
Need for CT Head Prior to LP?
Papilledema
Significant immunocompromise (eg. AIDS, transplant, neutropenia, chemotherapy, high dose glucocorticoids)
Focal neurological deficit except cranial nerve VI or VII palsy
History of CNS disease (e.g. mass lesion, prior stroke)
GCS <= 12
New onset seizures
Done
Amoxicillin-Clavulanate
C.diff Risk
Medium
Oral Bioavail.
Excellent
Cost
$2/day
Spectrum of Activity
General Spectrum of Activity
Antibiogram Data
Dosing
Adult
Renal
General Information

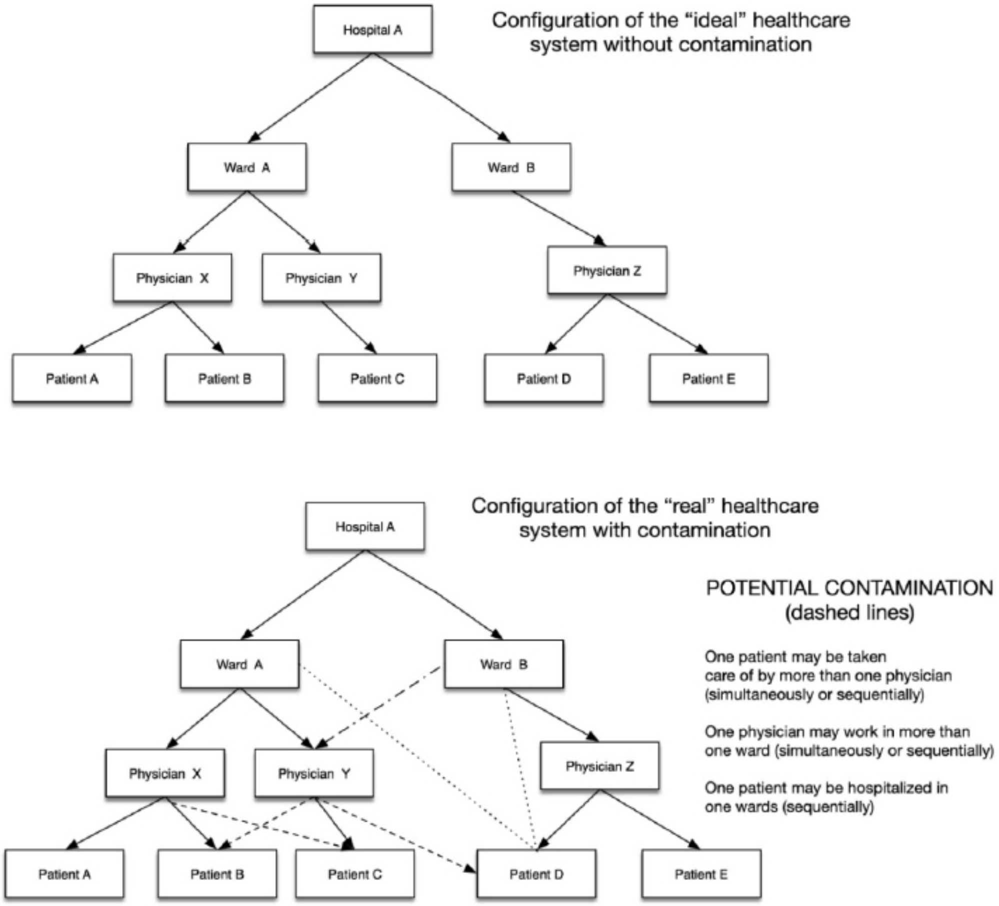
Configuration of the "ideal" healthcare system without contamination
Hospital A
Ward A
Ward B
Physician X
Physician Y
Physician Z
Patient A
Patient B
Patient C
Patient D
Patient E
Configuration of the "real" healthcare system with contamination
Hospital A
Ward A
Ward B
Physician X
Physician Y
Physician Z
Patient A
Patient B
Patient C
Patient D
Patient E
POTENTIAL CONTAMINATION (dashed lines)
One patient may be taken care of by more than one physician (simultaneously or sequentially)
One physician may work in more than one ward (simultaneously or sequentially)
One patient may be hospitalized in one wards (sequentially)

Appendix 1

CRF for appropriate antimicrobial drug use

1. Age of patient on date on study inclusion?
2. Gender of patient?
3. Allergy listed / specified?
    a. Listed & specified
    b. Listed without specification
    c. Not listed
    d. Not assessable
4. Date of hospital admission?
5. To which ward is patient admitted?
6. Date of admission to this ward unit?
7. Date of discharge from this ward/unit?
8. Indication listed or documented in admission note within 24 hours after starting antimicrobial drugs?

9.1.1 On which ward was the first antimicrobial drug prescribed?

9.1.2 What antimicrobial drug was prescribed?

9.1.3 On what date was the antimicrobial drug prescribed?

9.1.4 At what time was the antimicrobial drug prescribed?

9.1.5 Initial route of first antimicrobial drug prescribed?

9.1.6 Were there more than one therapeutic antimicrobial drug prescribed?

10. Correct choice of antimicrobial drugs according to the guidelines at time of prescribing according to indication?
    a. Fully concordant
    b. Partially concordant
    c. Not concordant
    d. No guideline available for specific indication
    e. Not assessable (no indication written)
11. Dosing regimen correct for all of the given antimicrobial drugs (adjusted for renal function too)?
12. Initial route of administration correct according to local guideline of all antimicrobial drugs?
13. Blood cultures performed?
14. Cultures or relevant microbial testing from suspected sites of infection performed according to local guideline (excluding blood cultures)?

For peer review only

15. PCR/Culture taken during this admission positive for C. difficile or MDRO (MRSA, ESBL-E, CPE, VRE, multidrug resistant P. aeruginosa)?
16. De-escalation from empirical to pathogen-directed therapy within 5 days after starting treatment for all antimicrobial drugs?
17. Switch IV to PO after 48-96h if clinically appropriate according to local guidelines?
18. Duration of antibiotic plan documented in medical chart? (between admission to this ward and day 5)
19. Duration of actual given antimicrobial drugs in accordance to local guidelines?
20. Therapeutic drug monitoring (TDM) according to local guidelines (aminoglycosides, glycopeptides, azoles) for all antimicrobial drugs?
21. Did the patient receive antimicrobial drugs <30 days before start of (empirical/targeted) treatment?
22. Have any of the following complications occurred?
    a. In-hospital mortality within 30 days after hospital admission (all cause)
    b. Transfer to intermediate care
    c. Transfer to ICU
    d. Unplanned readmission within 30 days after this admission
    e. Combination of answers above
    f. No
23. Is there an ID specialist / clinical microbiologist consulted for this treatment episode?
    a. Yes, in the first 5 days after start of antimicrobial treatment
    b. Yes, after 5 days of start antimicrobial treatment
    c. No
    d. Not assessable

For peer review only

Appendix 2: Post-intervention questionnaire

For each of the questions below, circle the response that characterizes best how you feel about each statement,

where: 1 = Strongly disagree, 2 = Disagree, 3 = Neither disagree nor agree, 4 = Agree, 5 = Strongly agree

	Strongly disagree	Disagree	Neither agree nor disagree	Agree	Strongly agree
1. This app explains clearly what I have to do and in which order	1	2	3	4	5
2. This app includes every step of appropriate antibiotic use in the hospital	1	2	3	4	5
3. This app is too complex for use in daily practice	1	2	3	4	5
4. Guidelines in the app were accessible	1	2	3	4	5
5. Guidelines in the app were consistent with other guidelines	1	2	3	4	5
6. This app fits in current practices	1	2	3	4	5
7. The benefits of using the app are clear	1	2	3	4	5
8. This app is feasible for all my patients who receive IV antibiotics	1	2	3	4	5
9. This app is a threat to my professional autonomy	1	2	3	4	5

For peer review only

10. I already knew the guidelines in the app	1	2	3	4	5
11. I trust the guidelines in the app	1	2	3	4	5
	Strongly disagree	Disagree	Neither agree nor disagree	Agree	Strongly agree
12. I agree with the guidelines in the app	1	2	3	4	5
13. I have a positive attitude towards guidelines in general	1	2	3	4	5
14. I expect that this app will improve the quality of my antibiotic prescriptions	1	2	3	4	5
15. I could not observe benefits of using the app	1	2	3	4	5
16. It is part of my job to use this app	1	2	3	4	5
17. I intend to use the app to assess the guidelines	1	2	3	4	5
18. I am capable of using this app	1	2	3	4	5
19. It does not take a lot of effort to use this app	1	2	3	4	5
20. I was able to try the app before using it for actual prescriptions	1	2	3	4	5
21. I have enough knowledge and expertise to use the app adequately	1	2	3	4	5

For peer review only

22. I have good previous experiences with working with an app	1	2	3	4	5
23. Colleagues will support me to use this	1	2	3	4	5
24. Supervisors will support me to use this app	1	2	3	4	5

	Strongly disagree	Disagree	Neither agree nor disagree	Agree	Strongly agree
25. Nurses will support me to use this app	1	2	3	4	5
26. Colleagues will use this app	1	2	3	4	5
27. Colleagues will expect me to use the app	1	2	3	4	5
28. Supervisors will expect me to use the app	1	2	3	4	5
29. Nurses will expect me to use the app	1	2	3	4	5
30. Patient will be satisfied that this app is being used	1	2	3	4	5
31. I expect that this app will improve the patient's antibiotic treatment	1	2	3	4	5
32. There are enough financial resources to use the app as it is meant to be used	1	2	3	4	5
33. I can use the app while working with the electronic health records	1	2	3	4	5

For peer review only

system					
34. External support is needed to implement the app on organizational level	1	2	3	4	5

For peer review only

SPIRIT — STANDARD PROTOCOL ITEMS: RECOMMENDATIONS FOR INTERVENTIONAL TRIALS

SPIRIT 2013 Checklist: Recommended items to address in a clinical trial protocol and related documents*

Section/item	Item No	Description	Addressed on page number
**Administrative information**			
Title	1	Descriptive title identifying the study design, population, interventions, and, if applicable, trial acronym	_____1_______
Trial registration	2a	Trial identifier and registry name. If not yet registered, name of intended registry	_____2_______
	2b	All items from the World Health Organization Trial Registration Data Set	_____1_______
Protocol version	3	Date and version identifier	______________
Funding	4	Sources and types of financial, material, and other support	_______13_____
Roles and responsibilities	5a	Names, affiliations, and roles of protocol contributors	_____1, 13____
	5b	Name and contact information for the trial sponsor	_______1______
	5c	Role of study sponsor and funders, if any, in study design; collection, management, analysis, and interpretation of data; writing of the report; and the decision to submit the report for publication, including whether they will have ultimate authority over any of these activities	______________
	5d	Composition, roles, and responsibilities of the coordinating centre, steering committee, endpoint adjudication committee, data management team, and other individuals or groups overseeing the trial, if applicable (see Item 21a for data monitoring committee)	______________

For peer review only - http://bmjopen.bmj.com/site/about/guidelines.xhtml

**Introduction**			
Background and rationale	6a	Description of research question and justification for undertaking the trial, including summary of relevant studies (published and unpublished) examining benefits and harms for each intervention	______3, 4_____
	6b	Explanation for choice of comparators	_____________
Objectives	7	Specific objectives or hypotheses	______4______
Trial design	8	Description of trial design including type of trial (eg, parallel group, crossover, factorial, single group), allocation ratio, and framework (eg, superiority, equivalence, noninferiority, exploratory)	______4______
**Methods: Participants, interventions, and outcomes**			
Study setting	9	Description of study settings (eg, community clinic, academic hospital) and list of countries where data will be collected. Reference to where list of study sites can be obtained	______4______
Eligibility criteria	10	Inclusion and exclusion criteria for participants. If applicable, eligibility criteria for study centres and individuals who will perform the interventions (eg, surgeons, psychotherapists)	______6______
Interventions	11a	Interventions for each group with sufficient detail to allow replication, including how and when they will be administered	______6______
	11b	Criteria for discontinuing or modifying allocated interventions for a given trial participant (eg, drug dose change in response to harms, participant request, or improving/worsening disease)	_____________
	11c	Strategies to improve adherence to intervention protocols, and any procedures for monitoring adherence (eg, drug tablet return, laboratory tests)	______7, 8____
	11d	Relevant concomitant care and interventions that are permitted or prohibited during the trial	______-______
Outcomes	12	Primary, secondary, and other outcomes, including the specific measurement variable (eg, systolic blood pressure), analysis metric (eg, change from baseline, final value, time to event), method of aggregation (eg, median, proportion), and time point for each outcome. Explanation of the clinical relevance of chosen efficacy and harm outcomes is strongly recommended	______7, 8____
Participant timeline	13	Time schedule of enrolment, interventions (including any run-ins and washouts), assessments, and visits for participants. A schematic diagram is highly recommended (see Figure)	__4, 5, Figure 1_

For peer review only - http://bmjopen.bmj.com/site/about/guidelines.xhtml

Sample size	14	Estimated number of participants needed to achieve study objectives and how it was determined, including clinical and statistical assumptions supporting any sample size calculations	_____10______
Recruitment	15	Strategies for achieving adequate participant enrolment to reach target sample size	_____10______
**Methods: Assignment of interventions (for controlled trials)**			
Allocation:			
Sequence generation	16a	Method of generating the allocation sequence (eg, computer-generated random numbers), and list of any factors for stratification. To reduce predictability of a random sequence, details of any planned restriction (eg, blocking) should be provided in a separate document that is unavailable to those who enrol participants or assign interventions	_____5_______
Allocation concealment mechanism	16b	Mechanism of implementing the allocation sequence (eg, central telephone; sequentially numbered, opaque, sealed envelopes), describing any steps to conceal the sequence until interventions are assigned	______5, 6____
Implementation	16c	Who will generate the allocation sequence, who will enrol participants, and who will assign participants to interventions	______5 ,6 ____
Blinding (masking)	17a	Who will be blinded after assignment to interventions (eg, trial participants, care providers, outcome assessors, data analysts), and how	______9______
	17b	If blinded, circumstances under which unblinding is permissible, and procedure for revealing a participant's allocated intervention during the trial	_____________
**Methods: Data collection, management, and analysis**			
Data collection methods	18a	Plans for assessment and collection of outcome, baseline, and other trial data, including any related processes to promote data quality (eg, duplicate measurements, training of assessors) and a description of study instruments (eg, questionnaires, laboratory tests) along with their reliability and validity, if known. Reference to where data collection forms can be found, if not in the protocol	_____7, 9_______
	18b	Plans to promote participant retention and complete follow-up, including list of any outcome data to be collected for participants who discontinue or deviate from intervention protocols	_____10______

Data management	19	Plans for data entry, coding, security, and storage, including any related processes to promote data quality (eg, double data entry; range checks for data values). Reference to where details of data management procedures can be found, if not in the protocol	_____7_______
Statistical methods	20a	Statistical methods for analysing primary and secondary outcomes. Reference to where other details of the statistical analysis plan can be found, if not in the protocol	______9, 10____
	20b	Methods for any additional analyses (eg, subgroup and adjusted analyses)	_____9, 10_____
	20c	Definition of analysis population relating to protocol non-adherence (eg, as randomised analysis), and any statistical methods to handle missing data (eg, multiple imputation)	______10______
**Methods: Monitoring**			
Data monitoring	21a	Composition of data monitoring committee (DMC); summary of its role and reporting structure; statement of whether it is independent from the sponsor and competing interests; and reference to where further details about its charter can be found, if not in the protocol. Alternatively, an explanation of why a DMC is not needed	______9______
	21b	Description of any interim analyses and stopping guidelines, including who will have access to these interim results and make the final decision to terminate the trial	_____________
Harms	22	Plans for collecting, assessing, reporting, and managing solicited and spontaneously reported adverse events and other unintended effects of trial interventions or trial conduct	_____________
Auditing	23	Frequency and procedures for auditing trial conduct, if any, and whether the process will be independent from investigators and the sponsor	_____________
**Ethics and dissemination**			
Research ethics approval	24	Plans for seeking research ethics committee/institutional review board (REC/IRB) approval	_____10______
Protocol amendments	25	Plans for communicating important protocol modifications (eg, changes to eligibility criteria, outcomes, analyses) to relevant parties (eg, investigators, REC/IRBs, trial participants, trial registries, journals, regulators)	_____________

For peer review only - http://bmjopen.bmj.com/site/about/guidelines.xhtml

Consent or assent	26a	Who will obtain informed consent or assent from potential trial participants or authorised surrogates, and how (see Item 32)	_____10______
	26b	Additional consent provisions for collection and use of participant data and biological specimens in ancillary studies, if applicable	_____________
Confidentiality	27	How personal information about potential and enrolled participants will be collected, shared, and maintained in order to protect confidentiality before, during, and after the trial	_____7_______
Declaration of interests	28	Financial and other competing interests for principal investigators for the overall trial and each study site	_____13______
Access to data	29	Statement of who will have access to the final trial dataset, and disclosure of contractual agreements that limit such access for investigators	_____________
Ancillary and post-trial care	30	Provisions, if any, for ancillary and post-trial care, and for compensation to those who suffer harm from trial participation	_____________
Dissemination policy	31a	Plans for investigators and sponsor to communicate trial results to participants, healthcare professionals, the public, and other relevant groups (eg, via publication, reporting in results databases, or other data sharing arrangements), including any publication restrictions	______10______
	31b	Authorship eligibility guidelines and any intended use of professional writers	_____________
	31c	Plans, if any, for granting public access to the full protocol, participant-level dataset, and statistical code	_____________
**Appendices**			
Informed consent materials	32	Model consent form and other related documentation given to participants and authorised surrogates	_____________
Biological specimens	33	Plans for collection, laboratory evaluation, and storage of biological specimens for genetic or molecular analysis in the current trial and for future use in ancillary studies, if applicable	_____________

*It is strongly recommended that this checklist be read in conjunction with the SPIRIT 2013 Explanation & Elaboration for important clarification on the items. Amendments to the protocol should be tracked and dated. The SPIRIT checklist is copyrighted by the SPIRIT Group under the Creative Commons "Attribution-NonCommercial-NoDerivs 3.0 Unported" license.

For peer review only - http://bmjopen.bmj.com/site/about/guidelines.xhtml

BMJ Open

Study protocol for an international, multicenter stepped-wedge cluster randomized trial to evaluate the impact of a digital antimicrobial stewardship smartphone application

Journal:	*BMJ Open*
Manuscript ID	bmjopen-2019-033640.R2
Article Type:	Protocol
Date Submitted by the Author:	24-Feb-2020
Complete List of Authors:	Helou, R.I.; Erasmus Medical Center, Department of Medical Microbiology and Infectious Diseases<br>Catho, Gaud ; Hopitaux Universitaires de Geneve, Department of Infectious Diseases<br>Peyravi Latif, Annabel; Uppsala University Hospital, Department of Infectious Diseases<br>Mouton, Johan; Erasmus Medical Center, Department of Medical Microbiology and Infectious Diseases<br>Hulscher, M; Radboud University Medical Center, Scientific Center for Quality of Healthcare (IQ healthcare), Radboud Institute for Health Sciences<br>Teerenstra, Steven; Radboud University Medical Center, Department for Health Evidence, Radboud Institute for Health Sciences<br>Conly, John; University of Calgary and Alberta Health Services, Department of Medicine<br>Huttner, Benedikt D; Hopitaux Universitaires de Geneve, Department of Infectious Diseases<br>Tängdén, Thomas; Uppsala University Hospital, Department of Infectious Diseases<br>Verbon, Annelies ; Erasmus Medical Center, Department of Medical Microbiology and Infectious Diseases
**Primary Subject Heading**:	Infectious diseases
Secondary Subject Heading:	Health informatics
Keywords:	INFECTIOUS DISEASES, MICROBIOLOGY, Protocols & guidelines < HEALTH SERVICES ADMINISTRATION & MANAGEMENT, Information technology < BIOTECHNOLOGY & BIOINFORMATICS, THERAPEUTICS

SCHOLARONE™
Manuscripts

I, the Submitting Author has the right to grant and does grant on behalf of all authors of the Work (as defined in the below author licence), an exclusive licence and/or a non-exclusive licence for contributions from authors who are: i) UK Crown employees; ii) where BMJ has agreed a CC-BY licence shall apply, and/or iii) in accordance with the terms applicable for US Federal Government officers or employees acting as part of their official duties; on a worldwide, perpetual, irrevocable, royalty-free basis to BMJ Publishing Group Ltd ("BMJ") its licensees and where the relevant Journal is co-owned by BMJ to the co-owners of the Journal, to publish the Work in this journal and any other BMJ products and to exploit all rights, as set out in our licence.

The Submitting Author accepts and understands that any supply made under these terms is made by BMJ to the Submitting Author unless you are acting as an employee on behalf of your employer or a postgraduate student of an affiliated institution which is paying any applicable article publishing charge ("APC") for Open Access articles. Where the Submitting Author wishes to make the Work available on an Open Access basis (and intends to pay the relevant APC), the terms of reuse of such Open Access shall be governed by a Creative Commons licence – details of these licences and which Creative Commons licence will apply to this Work are set out in our licence referred to above.

Other than as permitted in any relevant BMJ Author's Self Archiving Policies, I confirm this Work has not been accepted for publication elsewhere, is not being considered for publication elsewhere and does not duplicate material already published. I confirm all authors consent to publication of this Work and authorise the granting of this licence.

For peer review only

Study protocol for an international, multicenter stepped-wedge cluster randomized trial to evaluate the impact of a digital antimicrobial stewardship smartphone application

For peer review only

R.I. Helou[1], G. Catho[2], A. Peyravi Latif[3], Johan Mouton[1], M. Hulscher[4], S. Teerenstra[5], J. Conly[6], B. D. Huttner[2], T. Tängdén[3], A. Verbon[1*]

1) Department of Medical Microbiology and Infectious Diseases, Erasmus Medical Center, Rotterdam, The Netherlands
2) Department of Infectious Diseases, Geneva University Hospitals and Faculty of Medicine, Geneva, Switzerland
3) Department of Infectious Diseases, Uppsala University Hospital, Uppsala, Sweden
4) Scientific Center for Quality of Healthcare (IQ healthcare), Radboud Institute for Health Sciences, Radboud University Medical Center
5) Department for Health Evidence, Radboud Institute for Health Sciences, Radboud University Medical Center
6) Department of Medicine, University of Calgary and Alberta Health Services, Calgary, Alberta, Canada

*) corresponding author
email: a.verbon@erasmusmc.nl

Abstract

Introduction With the widespread use of electronic health records (EHR) and handheld electronic devices in hospitals, informatics-based antimicrobial stewardship (AMS) interventions hold great promise as tools to promote appropriate antimicrobial drug prescribing. However, more research is needed to evaluate their optimal design and impact on quantity and quality of antimicrobial prescribing.

Methods and analysis Use of smartphone-based digital stewardship applications (app) with local guideline directed empirical antimicrobial use by physicians will be compared to antimicrobial prescription as per usual as primary outcome in three hospitals in the Netherlands, Sweden and Switzerland. Secondary outcomes will include antimicrobial use metrics, clinical and process outcomes. A multicenter stepped-wedge cluster randomized trial will randomize entities defined as wards or specialty regarding time of introduction of the intervention. We will include 36 hospital entities with 7 measurement periods in which the primary outcome will be measured in 15 participating patients per time period per cluster. At participating wards, patients of at least 18 years of age using antimicrobials will be included. After a baseline period of 2-week measurements, 6 periods of 4 weeks will follow in which the intervention is introduced in 6 wards (in 3 hospitals) until all 36 wards have implemented the intervention. Thereafter, we allow use of the app by everyone, and evaluate the sustainability of the app use 6 months later.

Ethics and dissemination This protocol has been approved by the institutional review board (IRB) of each participating center. Results will be disseminated via media, to healthcare professionals via professional training and meetings and to researchers via conferences and publications.

Registration details This study is registered on ClinicalTrials.gov under identifier NCT03793946.

For peer review only

Strengths and limitations of this study

- This study will be an international randomized, multi-center, clinical trial using a rigorous design and methodology to evaluate the impact of an AMS smartphone app for the hospital setting
- The app can easily be adapted to local guidelines which is a key feature for potential future use in other settings
- The primary outcome is appropriate empiric antimicrobial therapy. The study is conducted in three hospitals with relatively high rates of appropriate antibiotic use, which may theoretically lead to a higher risk of a negative trial
- In this cluster randomized trial, wards/specialties are the unit of randomization and there is a risk of contamination through physicians changing between wards or among specialties, although this risk is lower than for a single-center randomized trial. Restricted access to the software and tracking of app use will be applied to monitor possible contamination.

Introduction

Antimicrobial drugs are an indispensable part of modern medicine, which depends on effective drugs for prophylaxis and treatment. Yet, only around 40-70% of empiric antimicrobial regimens are considered appropriate with regard to their indication, the choice of agent, dosing or duration.(1-4) Inappropriate antimicrobial use enhances the risks of treatment failure and side effects in the individual patient, and accelerates selection and transmission of antimicrobial-resistant pathogens in health care settings and the community.(5) Many antimicrobial stewardship (AMS) interventions have been shown to increase appropriateness of antimicrobial drug prescribing.(6)

A software application (app), which is compatible with mobile electronic devices such as smartphones and tablets, and increases the availability of existing national and local antibiotic guidelines plus local antibiograms, may improve antimicrobial prescribing, reduce medication errors and ultimately improve patient outcome. Due to its highly customizable nature, a digital app has the potential to become an effective AMS tool in all countries.

Mobile-software apps seem particularly interesting for the purpose of AMS, since they do not require access to electronic health records or computerized physician order entry systems, that are costly, difficult to maintain and not easily customizable. Most healthcare workers own a smartphone and often use it to access information to assist in their treatment decisions.(7, 8) The

For peer review only

vast majority of available apps developed to support prescription of medicines are limited to knowledge content and do not provide decision support algorithms. Further, they have rarely been evaluated in clinical trials.(9) To date, most reported digital AMS interventions are single-center studies with limited internal validity due to methodological limitations and uncertain external validity.(4, 10, 11)

In the present study, we will evaluate the implementation and impact of a smartphone-based software application (the AB-Assistant, Spectrum, Calgary, Canada) adapted to local conditions in an international clinical trial using a stepped-wedge cluster randomized design and appropriateness of empiric antimicrobial prescribing as the primary outcome.

Methods and analysis

Setting

This study will be conducted in three academic, tertiary care hospitals in Europe: Erasmus University Medical Center, Rotterdam, the Netherlands; Uppsala University Hospital, Uppsala, Sweden; and Geneva University Hospitals, Geneva, Switzerland (Table 1).

Study design

We plan to conduct an international, multicenter, stepped wedge cluster randomized trial to assess whether making a smartphone application for AMS (Spectrum) can improve the appropriateness of antimicrobial prescribing in hospitalized patients. Table 2 illustrates the study question according to the PICOT framework.

In total, 36 medical and surgical entities (defined as wards (Sweden) or specialties (Switzerland, The Netherlands) will be included in the study using a stepwise design with 6 entities (2 entities per participating hospital) 4-weekly at 6 introduction moments (Figure 1). The primary outcome is appropriateness of empiric antimicrobial prescriptions assessed prior to implementation and up to 12 months after the intervention (after 6 months free use of the app). Empiric therapy is defined as (1) treatment started based on clinical evaluation before culture results are known, (2) the duration of treatment is longer than 24 hours (from the moment of prescription) and (3) the treatment is not specifically recorded as prophylactic. Initial targeted therapy for which there is a local guideline available (e.g. C. difficile) may be assessed for appropriateness.

For peer review only

The entities will be randomized as to the time period of making the app available (see intervention below). The order of implementation will be determined by a computer-generated list of random numbers programmed by a statistician otherwise not involved in the trial and AMS activities. Randomization will be stratified by type of specialty (medicine, surgery etc.). After a baseline period of 2-week measurements, 6 periods of 4 weeks will follow in which the intervention is introduced in 6 entities (in 3 hospitals) until all entities have implemented the intervention. We plan to include 36 entities in total (see sample size calculation below) during the 6 introduction moments and at the end of the inclusion time we will allow the use of the app by everyone, also wards/specialties not included in the study. In this way, the trend in appropriate antimicrobial use can be monitored per ward/specialty and the decrease of antimicrobial use and appropriateness of empirical antimicrobial therapy can be followed during the stepwise implementation.(12) At 12 months (after 6 months free use of the app) we will have a 2-week measurement period to evaluate the sustainability of the intervention. The study will start between February 2020 and June 2020, and end 12 months later.

Justification of the stepped wedge cluster randomized study design

The stepped-wedge cluster randomized design was chosen after considering several advantages and disadvantages of randomization at the patient, physician, specialty and ward level. Patients are the "classic unit" of randomization and outcomes are often assessed at the patient level. However, there is a large risk of contamination of the intervention since one patient is often taken care of by many different physicians (see Figure 2). Furthermore, the study intervention targets physicians and not patients, therefore precluding randomization at the patient level as an option. Randomization at the physician-level is, however, equally problematic since prescribing decisions in hospitals are often made by teams of physicians, making it difficult to assign a decision to a specific individual physician and introducing the risk of contamination between physicians within a team. We therefore decided to use the wards/specialty as entity of randomization since it minimizes the risk of contamination and reflects "real-life" antimicrobial stewardship in hospitals where interventions are usually implemented at a ward/specialty level.

The stepped-wedge design, which involves the randomized and sequential rollout of the intervention to clusters over time, was chosen because it can model and adjust for underlying temporal trends and results in all clusters having access to the intervention at the end of the rollout period which seems desirable from an antimicrobial stewardship perspective.(13)

For peer review only

Study population

In our study, the intervention will be targeted at physicians that prescribe antimicrobials. To avoid possible contamination, the prescribers will be randomized at the ward/specialty level and not individually (see above). Process parameters will be determined in physicians who have been randomized to use the app per the process indicated above. Outcome parameters such as appropriate antimicrobial use will be determined at the patient level at participating wards (control and intervention) who meet the following criteria: being a medical or surgical specialty patient at least 18 years of age for whom antimicrobial drugs are prescribed.

Intervention

The Spectrum application, available for iOS and Android and on the web, has been developed in Canada (www.spectrum.app) and is now being used in over 50 hospitals, mainly in Canada and the United States of America. The app has been developed with the help of human factor experts and integrates multiple resources into a unified user-friendly interface for use at point-of-care to improve antimicrobial prescribing (Figure 3).

For the purpose of this study the app and the content management system were extended to also support languages other than English (French, Dutch, Swedish) and will be used for prescribing guidelines, pathogens, antimicrobial drugs and antibiograms. App content such as local guidelines, information regarding antimicrobial drugs, and micro-organisms of each hospital was entered by investigators of the Dutch, Swiss and Swedish hospitals (RH, GC, BH, APL). App content is identical to content of the local guidelines if these are published on a website. Minor discrepancies may occur if the local guidelines are published in a digital document (i.e. PDF) or on paper. The app can be more up to date in those cases.

The allocation of the timing to introduce the app will be concealed to the participating cluster up until 2 weeks before initiating use of the intervention. At the start of the intervention physicians on the intervention ward(s) will be informed about the function and content of the app. During the trial, only physicians on the intervention wards will be whitelisted to be able to use the app. Access to the app will be centrally withdrawn if a physician leaves the intervention ward (for example to work on a different (i.e. non-intervention) ward). The conventional route of guidelines (e.g. portable document formats (PDF), website or printed booklet) will still be available at all times. Physicians can freely choose between the app and the conventional route. Physicians on control wards will only be able to access the guidelines using the conventional route.

For peer review only

Patient and Public Involvement

No patients or public have been included in the study design.

Outcome parameters

Outcome parameters (Table 3) will be monitored using a digital CRF (Appendix 1) in the software application OpenClinica. (14)

Primary outcome parameter

Appropriateness of empiric antimicrobial therapy expressed as number and percentage of appropriate treatment episodes compared to all assessed treatment episodes.

Definitions for the primary outcome are:

- antimicrobial drugs assessed are ATC classes J01, J02, J03, J04, J05 (excluding anti-HIV drugs), P01, P02 and P03.
- treatment episodes are defined as the duration of treatment given for a specific infectious episode with gaps of treatment of no longer than one day.
- treatments are antimicrobials started during the measurement period at the participating entity. If a treatment was started within 24 hours before transfer in the emergency room or other ward and has not been changed during the first 24 hour stay at the ward, the treatment will be considered in accordance with the choice of the ward physician.
- treatments are in patients either newly admitted to the participating entities or already hospitalized during the pre-intervention period.
- appropriateness of empiric therapy includes choice, route and the dose of the antimicrobial drugs.
- appropriateness of therapy is in concordance with local treatment guidelines. If no guidelines are available, or if the treatment deviates from the guidelines or if treatment is guideline concordant but treatment should have deviated from the guideline (e.g. because of allergies, colonization with multidrug-resistant bacteria, other microbiological results, interactions, comorbidities or recent exposure to antimicrobials), anonymized cases will be discussed among 2 independent local infectious disease (ID) physicians. If they do not agree a third independent local ID physician will be consulted to adjudicate.
- for each patient only the first treatment episode during the measurement period will be evaluated. For patients treated for >1 infection (e.g. pneumonia and *Clostridium difficile* infection) appropriateness will be assessed for all infections combined (e.g. if

For peer review only

treatment is appropriate for *C. difficile* infection but inappropriate for pneumonia the treatment would be categorized as inappropriate).

Secondary outcome parameters

- Total prescription of antimicrobial drugs on the cluster-level expressed as DDD and DOT denominated by patient-days and admission; subcategories by type of antimicrobial (antibiotic, antifungal, etc.), WHO AWaRE categories and restricted antimicrobial drugs (Table 3).
- Clinical secondary outcomes: length of hospital stay, in hospital mortality and readmissions within 30 days after discharge, incidence/prevalence of key antimicrobial resistant (AMR) pathogens such as *C. difficile*.
- Process parameters of the intervention: penetration of the AB-assistant (total users and number of use of app), and uptake differences between centers.

Alongside the RCT, an uptake analysis will be performed to assess actual use of the app as well as an evaluation of use.

Uptake analysis

A number of parameters regarding use of the app will be collected and made available on a secure dashboard to authorized users at each site. These include number of monthly users and monthly sessions, number of times each antimicrobial, pathogen or clinical pathway screen is viewed, use by hospital, department, and medical specialism. These data will enable in-app behavior to be correlated with actual prescribing behavior on ward level and site-wide basis, thereby strengthening the association of the intervention to observed outcomes.

Evaluation of use

Insufficient user friendliness and lack of integration into the workflow are key impediments to the uptake of IT-based AMS interventions. Therefore, we will evaluate the physicians' experiences with the use of the app. After each introduction period all physicians of intervention wards will receive a questionnaire to collect information on experiences using the app. The questionnaire will be based on information from the literature (15), see Appendix 2.

Blinding during evaluation of outcome parameters

For peer review only

Given the nature of the intervention and the primary outcome assessed it will be difficult to blind the outcome assessor. To assure that assessment bias is limited we will have a subset of outcomes (10%) assessed by an independent investigator blinded to the study period.

Statistical methods

Baseline characteristics of patients (e.g. gender, age, allergies, co-morbidities), use of antimicrobial drugs, appropriate antimicrobial therapy, and other outcome parameters will be described by entity.

To mitigate possible differences in time trends and baseline characteristics at patient and center level, primary analysis will be by center and effect estimates will be combined over centers using meta-analytic methods. These primary analyses will employ multivariable multilevel logistic models with time periods as fixed effects to adjust for time trend and treatment as fixed effect to estimate the odds ratio of treatment. Entities (ward/specialty) will be included as a random effect to account for the correlation of patients within entities, assuming this correlation is independent of time period of including the patient. Moreover, covariates will be included to adjust for age, gender, hospital, and specialty (medical, surgical). Similar models but using (generalized) linear multilevel models will be used for continuous outcomes or incidence/rate outcomes.

Sensitivity analyses may include the following: adjusting for additional confounders if imbalance at baseline is present; investigating deviations from the assumption that the correlation of patients within entities is independent of time period of including the patient; estimating differences in percentage (instead of odds ratios) using a (generalized) linear multilevel models with identity link and binomial error distribution; incorporating the three centers in one model.

Subgroup analysis for specialty and other factors as appropriate will be conducted.

Sample size

By including 24 entities (wards/specialties) with 7 measurement periods (including one baseline period) in which the primary outcome (appropriate empiric antimicrobial drug use) will be measured in 15 participating patients per entity per time period and an assumed intra-entity correlation coefficient (ICC) of 0.1, the study has a power of 81% to detect an absolute improvement in appropriate antimicrobial use by 10 percentage points from of 60% to 70%.(16) To account for potential drop-outs or a lower inclusion rate, we aim to include 36 clusters in the three participating hospitals.

Ethics and dissemination

For peer review only

This protocol has been approved by the institutional review board (IRB) of each participating center. Ethical approval has been obtained from the Medical Ethics Committee (METC) Erasmus MC, Rotterdam, the Netherlands, number MEC-2019-0172, the Swedish Ethical Review Authority, Uppsala, Sweden, number 2019-05075 and from the Commission cantonale d'éthique de la recherche (CCER), Geneva, Switzerland, number 2019-01061. Physicians will be informed about the app without obligation to use it, furthermore analysis of usage will be anonymous, therefore a waiver for informed consent for physicians has been asked. However, participating physicians will be asked for a digital informed consent the first time they use the app. To monitor patient data, consent will be asked according to the country legislation and IRB mandate.

Dissemination

Results will be disseminated to healthcare professionals and researchers via presentations at national and international conferences and publications in peer-reviewed journals. Furthermore, we plan to have a workshop with interested healthcare workers after the end of the study to disseminate experiences and findings. The public will be informed through press releases. General project data and metadata will be included in the supplementary data of published articles if needed, and/or shared through university repositories.

Discussion and implications

To date, few IT-based AMS interventions have been evaluated in randomized clinical trials.(4, 10, 11) Clinical decision making by means of clinical decision support systems (CDSS) integrated in the EHR present the "gold standard" of IT-based antimicrobial stewardship. However their widespread use has been limited by some major obstacles: [1] EHRs are still not universally implemented in all hospitals in high-income countries, and even less so in low- and middle income countries [2] even if an EHR is implemented it usually lacks essential components for CDSS such as a computerized physician order entry system [3] EHRs - especially those of commercial vendors – have designs that are difficult to modify/ adapt. By developing an "app" as a software program running on smartphones and other mobile devices such as tablets, an intermediate or complementary step to full CDSS may be offered. Spectrum is a smartphone-based digital stewardship application that is customizable to local guidelines by local AMS teams. In our stepped-wedge cluster randomized trial we will evaluate the impact of this app on appropriate antimicrobial prescribing in high income countries. However, smartphone adoption is consistently strong in middle income countries such as in the Caribbean and reached low-income countries in Africa.(17, 18) More and more healthcare workers have their own electronic devices (smartphone

For peer review only

or tablets).(7) AMS smartphone-based interventions can be made easily available, in high, low and middle income countries. The ability of this app will make antibiotic prescription guidelines more accessible for physicians all over the world, thereby helping to fight AMR.

References

1. van den Bosch CM, Hulscher ME, Natsch S, Wille J, Prins JM, Geerlings SE. Applicability of generic quality indicators for appropriate antibiotic use in daily hospital practice: a cross-sectional point-prevalence multicenter study. Clin Microbiol Infect. 2016;22(10):888 e1-e9.
2. Yong MK, Buising KL, Cheng AC, Thursky KA. Improved susceptibility of Gram-negative bacteria in an intensive care unit following implementation of a computerized antibiotic decision support system. J Antimicrob Chemother. 2010;65(5):1062-9.
3. McGregor JC, Weekes E, Forrest GN, Standiford HC, Perencevich EN, Furuno JP, et al. Impact of a computerized clinical decision support system on reducing inappropriate antimicrobial use: a randomized controlled trial. J Am Med Inform Assoc. 2006;13(4):378-84.
4. Baysari MT, Lehnbom EC, Li L, Hargreaves A, Day RO, Westbrook JI. The effectiveness of information technology to improve antimicrobial prescribing in hospitals: A systematic review and meta-analysis. Int J Med Inform. 2016;92:15-34.
5. Bell BG, Schellevis F, Stobberingh E, Goossens H, Pringle M. A systematic review and meta-analysis of the effects of antibiotic consumption on antibiotic resistance. BMC Infect Dis. 2014;14:13.
6. Davey P, Brown E, Charani E, Fenelon L, Gould IM, Holmes A, et al. Interventions to improve antibiotic prescribing practices for hospital inpatients. Cochrane Database Syst Rev. 2013(4):CD003543.
7. Patel RK, Sayers AE, Patrick NL, Hughes K, Armitage J, Hunter IA. A UK perspective on smartphone use amongst doctors within the surgical profession. Ann Med Surg (Lond). 2015;4(2):107-12.
8. Stergiannis P, Intas G, Toulia G, Tsolakoglou I, Kostagiolas P, Christodoulou E, et al. Clinical Use of Smartphones Among Medical and Nursing Staff in Greece: A Survey. Comput Inform Nurs. 2017;35(9):483-8.
9. Haffey F, Brady RR, Maxwell S. Smartphone apps to support hospital prescribing and pharmacology education: a review of current provision. Br J Clin Pharmacol. 2014;77(1):31-8.
10. Cresswell K, Mozaffar H, Shah S, Sheikh A. Approaches to promoting the appropriate use of antibiotics through hospital electronic prescribing systems: a scoping review. Int J Pharm Pract. 2017;25(1):5-17.
11. Rawson TM, Moore LSP, Hernandez B, Charani E, Castro-Sanchez E, Herrero P, et al. A systematic review of clinical decision support systems for antimicrobial management: are we failing to investigate these interventions appropriately? Clin Microbiol Infect. 2017;23(8):524-32.

For peer review only

12. Palmay L, Elligsen M, Walker SA, Pinto R, Walker S, Einarson T, et al. Hospital-wide rollout of antimicrobial stewardship: a stepped-wedge randomized trial. Clin Infect Dis. 2014;59(6):867-74.
13. Brown CA, Lilford RJ. The stepped wedge trial design: a systematic review. BMC Med Res Methodol. 2006;6:54.
14. OpenClinica LLC and collaborators. OpenClinica open source software, version 3.1.
15. Flottorp SA, Oxman AD, Krause J, Musila NR, Wensing M, Godycki-Cwirko M, et al. A checklist for identifying determinants of practice: a systematic review and synthesis of frameworks and taxonomies of factors that prevent or enable improvements in healthcare professional practice. Implement Sci. 2013;8:35.
16. Hussey MA, Hughes JP. Design and analysis of stepped wedge cluster randomized trials. Contemp Clin Trials. 2007;28(2):182-91.
17. GSMA. The Mobile Economy Latin America and the Caribbean 2018. 2018.
18. GSMA. The Mobile Economy Sub-Saharan Africa 2019. 2019.
19. World Health Organization. WHO Model Lists of Essential Medicines - EML – 20th edition. 2017.

For peer review only

For peer review only

Authors' contributions

A draft protocol was written by A. Verbon, B.D. Huttner, T. Tängdén, J. Conly and Johan Mouton. During extensive calls and meetings with A. Verbon, B.D. Huttner, T. Tängdén, J. Conly and Johan Mouton, R.I. Helou, G. Catho, A. Peyravi Latif, M. Hulscher and S. Teerenstra the introduction, methods and analysis, outcome parameters, evaluation of use, blinding during evaluation of outcome parameters, statistical methods, ethics and dissemination and, discussion and implications were further shaped and fine-tuned.

Funding statement

This work is supported by Joint Programming Initiative on Antimicrobial Resistance (JPIAMR), grant number JPIAMR2017-045.

Competing interests statement

None declared.

Table 1: characteristics of the participating centers

**Name**	**Geneva University Hospitals**	**Erasmus MC Rotterdam**	**Uppsala University Hospital**
**Website**	www.hug-ge.ch/en/	https://www.erasmusmc.nl/	https://www.akademiska.se/en/
**Abbreviation**	HUG	EMC	UUH
**City**	Geneva	Rotterdam	Uppsala
**Country**	Switzerland	The Netherlands	Sweden
**Care level**	Primary and Tertiary Care	Tertiary Care	Tertiary Care

For peer review only

**Academic affiliation**	Yes	Yes	Yes
**Number of beds**	About 1'900	900	About 1´000
**Availability of antibiotic guidelines**	Paper format and PDF<br>Updated every 2 years<br>Integrated into the EHR in some units in the context of a different study (Trial registration number: NCT03120975); COMPASS units not participating in this study	Website containing local guidelines. Updated every 2 years. Guidelines are based on the national guidelines of SWAB	Local guidelines in paper format and online (pdf). Updated every 2-3 years. National guidelines provided by STRAMA available online and in app format, not routinely used at UUH.
**Current standard of care antimicrobial stewardship**	• On demand ID consultations<br>• Review of all positive blood cultures<br>• Daily rounds on some units<br>• Approval required for certain antibiotics	• On demand ID consultations<br>• Daily rounds on Intensive care units, weekly rounds on all units<br>• Review and feedback for certain antibiotics<br>• Approval required for certain antibiotics<br>• Review of all positive blood cultures	• On demand ID consultations<br>• Daily rounds on intensive care units<br>• Adapted information and feedback to physicians at major departments 1-2 times per year

444

445

446

447

For peer review only

Table 2: PICOT of the study question

Population	Physicians involved in antimicrobial prescribing decisions for hospitalized adult patients in the participating centers
Intervention	Making a smartphone application with antimicrobial treatment recommendations available to physicians mentioned above
Comparator	Standard-of-care antimicrobial stewardship
Outcome	Appropriateness of empiric antimicrobial prescribing based on predefined criteria
Time	12 months consisting of a 6 months introduction period with 6 months follow-up to assess sustainability

For peer review only

465
466
467
468
469
470
471
472
473
474

475 **Table 3. Secondary study parameters/endpoints and other study parameters**

Outcome type	Examples	Data source
**Quantitative antimicrobial use**	• Total prescription of antimicrobial drugs1 in DDD/admission, DDD/PD, DOT/PD and DOT/admission<br>• Total prescription of broad spectrum and restricted antimicrobial drugs in DDD/admission<br>• Total prescription of antimicrobial drugs per AWaRE category(19) in DDD/admission<br>• Antimicrobial costs	• EHR<br>• Administrative data
**Patient Related**	• Length of hospital stay<br>• In hospital mortality within 30 days after admission (all cause)<br>• Unplanned hospital readmissions within 30 days after discharge<br>• Transfer to intermediate care or ICU within 30 days after admission	• EHR<br>• Administrative data

For peer review only

**Microbiologic and HAI**	• Incidence of healthcare facility onset *Clostridium difficile*<br>• Incidence clinical cultures with multi-drug resistant organisms (MRSA, ESBL-E, CPE, VRE, multidrug resistant *P. aeruginosa*) denominated per 1000 PD and admission	• EHR<br>• Microbiology database<br>• Infection control surveillance data
**Physician related**	• Uptake of the AB-assistant (total users and number of sessions per user, time spent per session, time spent per screen, number of times each screen is viewed)<br>• Differences in uptake between centers<br>• Actual use of app and experiences while using it	• Content Management System<br>• Content Management System<br>• Survey
**Other outcomes**	• Number of infectious diseases consultations	• EHR

DDD: defined daily dose **EHR:** electronic health record; **PD:** patient- days; **HAI**: hospital acquired infections; **CMS:** content management system'; **ICU:** intensive care unit; **MRSA:** methicillin resistant staphylococcus aureus; **ESBL-E:** extended-spectrum beta-lactamase-producing E; **CPE:** carbapenemase-producing enterobacteriaceae; **CRE:** carbapenem-resistant enterobacteriaceae

1 Antimicrobials belonging to Anatomical Therapeutic Chemical Classification System class J01, J02, J03, J04, J05 (excluding anti-HIV drugs), P01, P02 and P03, oral vancomycin (A07AA09) and fidaxomicin (A07AA12).

For peer review only

Figure legends

Figure 1 - Study design

In all time periods (total of 7) there is an uptake period of 2 days and a measure period of 26 days. During the baseline period only a measure period of 2 weeks will be performed.

Figure 2 - Interface of the AB-assistant app

Interface of the AB-assistant app. On the left the Home screen is shown. In the middle checkboxes are shown to guide the user to the correct therapy for Meningitis. The right screen shows details about Amoxicillin-Clavulanate regarding dosing and general spectrum of activity.

Figure 3 - Scheme of potential contamination

The potential contamination in a hospital, in and between wards, physicians and patients.

For peer review only

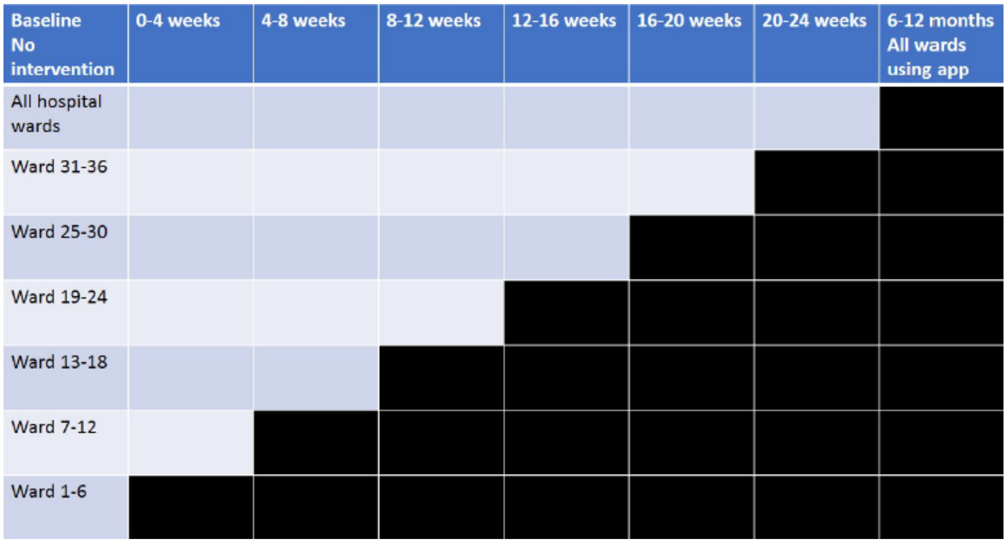
Baseline No intervention
0-4 weeks
4-8 weeks
8-12 weeks
12-16 weeks
16-20 weeks
20-24 weeks
6-12 months All wards using app
All hospital wards
Ward 31-36
Ward 25-30
Ward 19-24
Ward 13-18
Ward 7-12
Ward 1-6

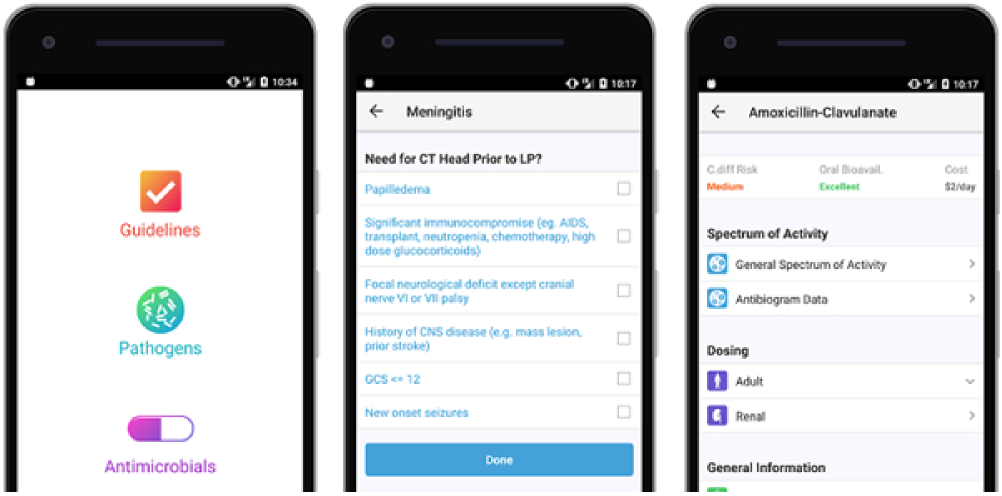
10:34
Guidelines
Pathogens
Antimicrobials
10:17
Meningitis
Need for CT Head Prior to LP?
Papilledema
Significant immunocompromise (eg. AIDS, transplant, neutropenia, chemotherapy, high dose glucocorticoids)
Focal neurological deficit except cranial nerve VI or VII palsy
History of CNS disease (e.g. mass lesion, prior stroke)
GCS <= 12
New onset seizures
Done
10:17
Amoxicillin-Clavulanate
C.diff Risk
Medium
Oral Bioavail.
Excellent
Cost
$2/day
Spectrum of Activity
General Spectrum of Activity
Antibiogram Data
Dosing
Adult
Renal
General Information

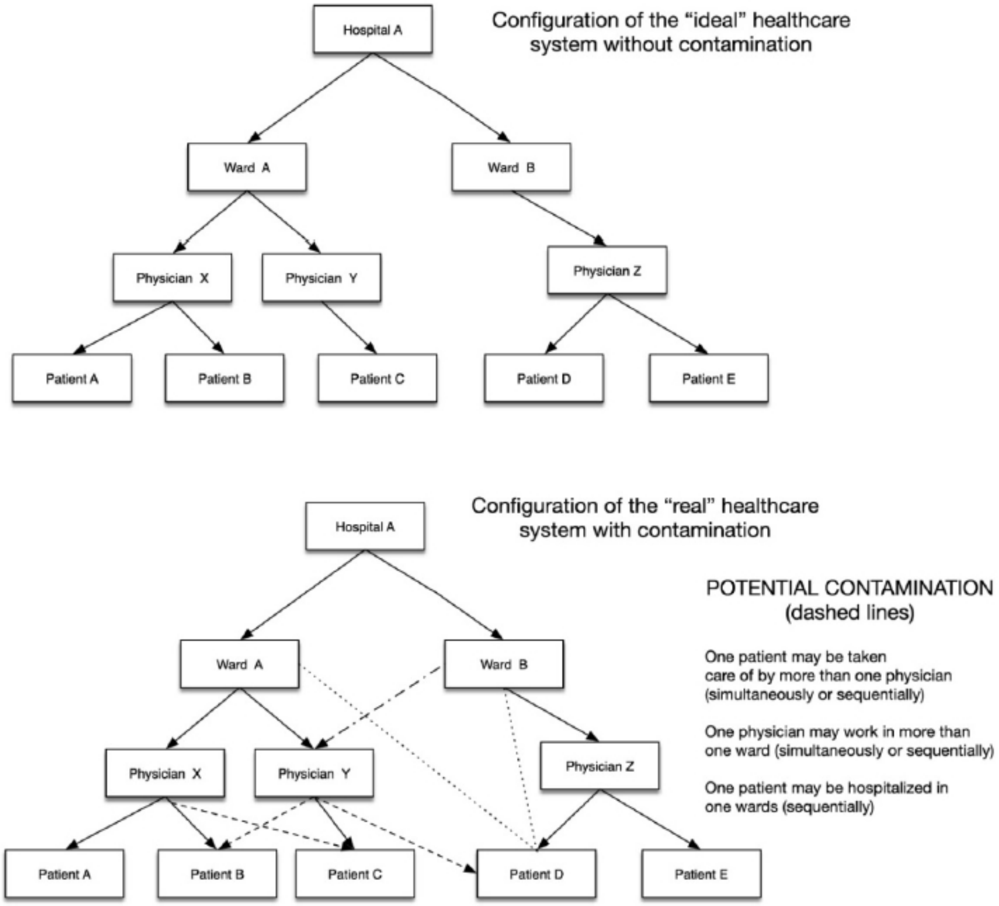
Hospital A
Configuration of the "ideal" healthcare system without contamination
Ward A
Ward B
Physician X
Physician Y
Physician Z
Patient A
Patient B
Patient C
Patient D
Patient E
Hospital A
Configuration of the "real" healthcare system with contamination
POTENTIAL CONTAMINATION (dashed lines)
One patient may be taken care of by more than one physician (simultaneously or sequentially)
One physician may work in more than one ward (simultaneously or sequentially)
One patient may be hospitalized in one wards (sequentially)
Ward A
Ward B
Physician X
Physician Y
Physician Z
Patient A
Patient B
Patient C
Patient D
Patient E

Appendix 1

CRF for appropriate antimicrobial drug use

1. Age of patient on date on study inclusion?
2. Gender of patient?
3. Allergy listed / specified?
    a. Listed & specified
    b. Listed without specification
    c. Not listed
    d. Not assessable
4. Date of hospital admission?
5. To which ward is patient admitted?
6. Date of admission to this ward unit?
7. Date of discharge from this ward/unit?
8. Indication listed or documented in admission note within 24 hours after starting antimicrobial drugs?

9.1.1 On which ward was the first antimicrobial drug prescribed?

9.1.2 What antimicrobial drug was prescribed?

9.1.3 On what date was the antimicrobial drug prescribed?

9.1.4 At what time was the antimicrobial drug prescribed?

9.1.5 Initial route of first antimicrobial drug prescribed?

9.1.6 Were there more than one therapeutic antimicrobial drug prescribed?

10. Correct choice of antimicrobial drugs according to the guidelines at time of prescribing according to indication?
    a. Fully concordant
    b. Partially concordant
    c. Not concordant
    d. No guideline available for specific indication
    e. Not assessable (no indication written)
11. Dosing regimen correct for all of the given antimicrobial drugs (adjusted for renal function too)?
12. Initial route of administration correct according to local guideline of all antimicrobial drugs?
13. Blood cultures performed?
14. Cultures or relevant microbial testing from suspected sites of infection performed according to local guideline (excluding blood cultures)?

For peer review only

15. PCR/Culture taken during this admission positive for C. difficile or MDRO (MRSA, ESBL-E, CPE, VRE, multidrug resistant P. aeruginosa)?
16. De-escalation from empirical to pathogen-directed therapy within 5 days after starting treatment for all antimicrobial drugs?
17. Switch IV to PO after 48-96h if clinically appropriate according to local guidelines?
18. Duration of antibiotic plan documented in medical chart? (between admission to this ward and day 5)
19. Duration of actual given antimicrobial drugs in accordance to local guidelines?
20. Therapeutic drug monitoring (TDM) according to local guidelines (aminoglycosides, glycopeptides, azoles) for all antimicrobial drugs?
21. Did the patient receive antimicrobial drugs <30 days before start of (empirical/targeted) treatment?
22. Have any of the following complications occurred?
    a. In-hospital mortality within 30 days after hospital admission (all cause)
    b. Transfer to intermediate care
    c. Transfer to ICU
    d. Unplanned readmission within 30 days after this admission
    e. Combination of answers above
    f. No
23. Is there an ID specialist / clinical microbiologist consulted for this treatment episode?
    a. Yes, in the first 5 days after start of antimicrobial treatment
    b. Yes, after 5 days of start antimicrobial treatment
    c. No
    d. Not assessable

For peer review only

Appendix 2: Post-intervention questionnaire

For each of the questions below, circle the response that characterizes best how you feel about each statement,

where: 1 = Strongly disagree, 2 = Disagree, 3 = Neither disagree nor agree, 4 = Agree, 5 = Strongly agree

	Strongly disagree	Disagree	Neither agree nor disagree	Agree	Strongly agree
1. This app explains clearly what I have to do and in which order	1	2	3	4	5
2. This app includes every step of appropriate antibiotic use in the hospital	1	2	3	4	5
3. This app is too complex for use in daily practice	1	2	3	4	5
4. Guidelines in the app were accessible	1	2	3	4	5
5. Guidelines in the app were consistent with other guidelines	1	2	3	4	5
6. This app fits in current practices	1	2	3	4	5
7. The benefits of using the app are clear	1	2	3	4	5
8. This app is feasible for all my patients who receive IV antibiotics	1	2	3	4	5
9. This app is a threat to my professional autonomy	1	2	3	4	5

For peer review only

10. I already knew the guidelines in the app	1	2	3	4	5
11. I trust the guidelines in the app	1	2	3	4	5
	Strongly disagree	Disagree	Neither agree nor disagree	Agree	Strongly agree
12. I agree with the guidelines in the app	1	2	3	4	5
13. I have a positive attitude towards guidelines in general	1	2	3	4	5
14. I expect that this app will improve the quality of my antibiotic prescriptions	1	2	3	4	5
15. I could not observe benefits of using the app	1	2	3	4	5
16. It is part of my job to use this app	1	2	3	4	5
17. I intend to use the app to assess the guidelines	1	2	3	4	5
18. I am capable of using this app	1	2	3	4	5
19. It does not take a lot of effort to use this app	1	2	3	4	5
20. I was able to try the app before using it for actual prescriptions	1	2	3	4	5
21. I have enough knowledge and expertise to use the app adequately	1	2	3	4	5

For peer review only

22. I have good previous experiences with working with an app	1	2	3	4	5
23. Colleagues will support me to use this	1	2	3	4	5
24. Supervisors will support me to use this app	1	2	3	4	5

	Strongly disagree	Disagree	Neither agree nor disagree	Agree	Strongly agree
25. Nurses will support me to use this app	1	2	3	4	5
26. Colleagues will use this app	1	2	3	4	5
27. Colleagues will expect me to use the app	1	2	3	4	5
28. Supervisors will expect me to use the app	1	2	3	4	5
29. Nurses will expect me to use the app	1	2	3	4	5
30. Patient will be satisfied that this app is being used	1	2	3	4	5
31. I expect that this app will improve the patient's antibiotic treatment	1	2	3	4	5
32. There are enough financial resources to use the app as it is meant to be used	1	2	3	4	5
33. I can use the app while working with the electronic health records	1	2	3	4	5

For peer review only

system					
34. External support is needed to implement the app on organizational level	1	2	3	4	5

For peer review only

STANDARD PROTOCOL ITEMS: RECOMMENDATIONS FOR INTERVENTIONAL TRIALS

SPIRIT 2013 Checklist: Recommended items to address in a clinical trial protocol and related documents*

Section/item	Item No	Description	Addressed on page number
**Administrative information**			
Title	1	Descriptive title identifying the study design, population, interventions, and, if applicable, trial acronym	1
Trial registration	2a	Trial identifier and registry name. If not yet registered, name of intended registry	2
	2b	All items from the World Health Organization Trial Registration Data Set	1
Protocol version	3	Date and version identifier	
Funding	4	Sources and types of financial, material, and other support	13
Roles and responsibilities	5a	Names, affiliations, and roles of protocol contributors	1, 13
	5b	Name and contact information for the trial sponsor	1
	5c	Role of study sponsor and funders, if any, in study design; collection, management, analysis, and interpretation of data; writing of the report; and the decision to submit the report for publication, including whether they will have ultimate authority over any of these activities	
	5d	Composition, roles, and responsibilities of the coordinating centre, steering committee, endpoint adjudication committee, data management team, and other individuals or groups overseeing the trial, if applicable (see Item 21a for data monitoring committee)	

For peer review only

Introduction

Background and rationale	6a	Description of research question and justification for undertaking the trial, including summary of relevant studies (published and unpublished) examining benefits and harms for each intervention	______3, 4_____
	6b	Explanation for choice of comparators	_____________
Objectives	7	Specific objectives or hypotheses	______4______
Trial design	8	Description of trial design including type of trial (eg, parallel group, crossover, factorial, single group), allocation ratio, and framework (eg, superiority, equivalence, noninferiority, exploratory)	______4______

Methods: Participants, interventions, and outcomes

Study setting	9	Description of study settings (eg, community clinic, academic hospital) and list of countries where data will be collected. Reference to where list of study sites can be obtained	______4______
Eligibility criteria	10	Inclusion and exclusion criteria for participants. If applicable, eligibility criteria for study centres and individuals who will perform the interventions (eg, surgeons, psychotherapists)	______6______
Interventions	11a	Interventions for each group with sufficient detail to allow replication, including how and when they will be administered	______6______
	11b	Criteria for discontinuing or modifying allocated interventions for a given trial participant (eg, drug dose change in response to harms, participant request, or improving/worsening disease)	_____________
	11c	Strategies to improve adherence to intervention protocols, and any procedures for monitoring adherence (eg, drug tablet return, laboratory tests)	______7, 8____
	11d	Relevant concomitant care and interventions that are permitted or prohibited during the trial	______-______
Outcomes	12	Primary, secondary, and other outcomes, including the specific measurement variable (eg, systolic blood pressure), analysis metric (eg, change from baseline, final value, time to event), method of aggregation (eg, median, proportion), and time point for each outcome. Explanation of the clinical relevance of chosen efficacy and harm outcomes is strongly recommended	______7, 8____
Participant timeline	13	Time schedule of enrolment, interventions (including any run-ins and washouts), assessments, and visits for participants. A schematic diagram is highly recommended (see Figure)	__4, 5, Figure 1_

Sample size	14	Estimated number of participants needed to achieve study objectives and how it was determined, including clinical and statistical assumptions supporting any sample size calculations	_____10______
Recruitment	15	Strategies for achieving adequate participant enrolment to reach target sample size	_____10______

Methods: Assignment of interventions (for controlled trials)

Allocation:

Sequence generation	16a	Method of generating the allocation sequence (eg, computer-generated random numbers), and list of any factors for stratification. To reduce predictability of a random sequence, details of any planned restriction (eg, blocking) should be provided in a separate document that is unavailable to those who enrol participants or assign interventions	_____5_______
Allocation concealment mechanism	16b	Mechanism of implementing the allocation sequence (eg, central telephone; sequentially numbered, opaque, sealed envelopes), describing any steps to conceal the sequence until interventions are assigned	______5, 6____
Implementation	16c	Who will generate the allocation sequence, who will enrol participants, and who will assign participants to interventions	______5 ,6 ____
Blinding (masking)	17a	Who will be blinded after assignment to interventions (eg, trial participants, care providers, outcome assessors, data analysts), and how	______9______
	17b	If blinded, circumstances under which unblinding is permissible, and procedure for revealing a participant's allocated intervention during the trial	_____________

Methods: Data collection, management, and analysis

Data collection methods	18a	Plans for assessment and collection of outcome, baseline, and other trial data, including any related processes to promote data quality (eg, duplicate measurements, training of assessors) and a description of study instruments (eg, questionnaires, laboratory tests) along with their reliability and validity, if known. Reference to where data collection forms can be found, if not in the protocol	_____7, 9_______
	18b	Plans to promote participant retention and complete follow-up, including list of any outcome data to be collected for participants who discontinue or deviate from intervention protocols	_____10______

Data management	19	Plans for data entry, coding, security, and storage, including any related processes to promote data quality (eg, double data entry; range checks for data values). Reference to where details of data management procedures can be found, if not in the protocol	_____7_______
Statistical methods	20a	Statistical methods for analysing primary and secondary outcomes. Reference to where other details of the statistical analysis plan can be found, if not in the protocol	______9, 10____
	20b	Methods for any additional analyses (eg, subgroup and adjusted analyses)	_____9, 10_____
	20c	Definition of analysis population relating to protocol non-adherence (eg, as randomised analysis), and any statistical methods to handle missing data (eg, multiple imputation)	______10______
**Methods: Monitoring**			
Data monitoring	21a	Composition of data monitoring committee (DMC); summary of its role and reporting structure; statement of whether it is independent from the sponsor and competing interests; and reference to where further details about its charter can be found, if not in the protocol. Alternatively, an explanation of why a DMC is not needed	______9______
	21b	Description of any interim analyses and stopping guidelines, including who will have access to these interim results and make the final decision to terminate the trial	_____________
Harms	22	Plans for collecting, assessing, reporting, and managing solicited and spontaneously reported adverse events and other unintended effects of trial interventions or trial conduct	_____________
Auditing	23	Frequency and procedures for auditing trial conduct, if any, and whether the process will be independent from investigators and the sponsor	_____________
**Ethics and dissemination**			
Research ethics approval	24	Plans for seeking research ethics committee/institutional review board (REC/IRB) approval	_____10______
Protocol amendments	25	Plans for communicating important protocol modifications (eg, changes to eligibility criteria, outcomes, analyses) to relevant parties (eg, investigators, REC/IRBs, trial participants, trial registries, journals, regulators)	_____________

For peer review only - http://bmjopen.bmj.com/site/about/guidelines.xhtml

Consent or assent	26a	Who will obtain informed consent or assent from potential trial participants or authorised surrogates, and how (see Item 32)	_____10______
	26b	Additional consent provisions for collection and use of participant data and biological specimens in ancillary studies, if applicable	_____________
Confidentiality	27	How personal information about potential and enrolled participants will be collected, shared, and maintained in order to protect confidentiality before, during, and after the trial	_____7_______
Declaration of interests	28	Financial and other competing interests for principal investigators for the overall trial and each study site	_____13______
Access to data	29	Statement of who will have access to the final trial dataset, and disclosure of contractual agreements that limit such access for investigators	_____________
Ancillary and post-trial care	30	Provisions, if any, for ancillary and post-trial care, and for compensation to those who suffer harm from trial participation	_____________
Dissemination policy	31a	Plans for investigators and sponsor to communicate trial results to participants, healthcare professionals, the public, and other relevant groups (eg, via publication, reporting in results databases, or other data sharing arrangements), including any publication restrictions	______10______
	31b	Authorship eligibility guidelines and any intended use of professional writers	_____________
	31c	Plans, if any, for granting public access to the full protocol, participant-level dataset, and statistical code	_____________
**Appendices**			
Informed consent materials	32	Model consent form and other related documentation given to participants and authorised surrogates	_____________
Biological specimens	33	Plans for collection, laboratory evaluation, and storage of biological specimens for genetic or molecular analysis in the current trial and for future use in ancillary studies, if applicable	_____________

*It is strongly recommended that this checklist be read in conjunction with the SPIRIT 2013 Explanation & Elaboration for important clarification on the items. Amendments to the protocol should be tracked and dated. The SPIRIT checklist is copyrighted by the SPIRIT Group under the Creative Commons "Attribution-NonCommercial-NoDerivs 3.0 Unported" license.

For peer review only - http://bmjopen.bmj.com/site/about/guidelines.xhtml
